# Supplementary material for: Quasi Pd1Ni single-atom surface alloy catalyst enables hydrogenation of nitriles to secondary amines
Source: Nat Commun. 2019 Nov 1;10:4998. doi: 10.1038/s41467-019-12993-x (PMC6825208; doi:10.1038/s41467-019-12993-x)
Supplement: Supplementary file 1 — Supplementary Info [file 41467_2019_12993_MOESM1_ESM.pdf]

Supplementary Information for

Quasi Pd_1Ni single-atom surface alloy catalyst enables hydrogenation of nitriles to secondary amines

Wang et al.

Supplementary Methods

Chemicals and Materials

Nickel nitrate hexahydrate ($Ni(NO_3)_2 \cdot 6H_2O$), palladium acetylacetonate ($Pd(acac)_2$, 99.5%), ammonium hydroxide ($NH_3 \cdot H_2O$, 26~28 wt%) and all solvents were purchased from Sinopharm Chemical Reagent Co. Ltd. (Shanghai, China). All nitrile substrates have analytical grade, and purchased from Aladdin Chemicals. The ALD precursors, palladium hexafluoroacetylacetate ($Pd(hfac)_2$, 99%), trimethyl(methylcyclo-pentadienyl)-platinum(IV) ($MeCpPtMe_3$, 99%) were purchased from Nanjing MO Yuan Scientific Instruments & Materials Co., Ltd. (Nanjing, China). The spherical SiO_2 support were synthesized according to the modified Stöber method[1]. Spherical Al_2O_3 support (99.5%; NANODUR) was purchased from Alfa Aesar. All gases were purchased from Nanjing Special Gases (Hefei, China). All chemicals were used as received without further purification.

Catalysts preparation

Preparation of Ni/SiO_2

Ni/SiO_2 catalyst was synthesized using the deposition-precipitation (DP) method. Typically, 0.63 g $Ni(NO_3)_2 \cdot 6H_2O$ was first dissolved in 50 mL deionized water. Then 0.4 g spherical SiO_2 was added into the solution and mixed under vigorous stirring at 50 °C for 0.5 h. Ammonia was added dropwise to adjust the pH value between 8 and 9. Then, the system was continued vigorously stirred for another 3 h. The precipitate was centrifuged and washed with deionized water and dried at 60 °C overnight. Finally, the dried material was calcined in 10% O_2 in Ar at 600 °C for 4 h, then reduced at the same temperature for another 3 h in 10% H_2 in Ar. The Ni loading was measured as 19 wt% using ICP-AES analysis.

Pd ALD: Synthesis of various $Pd-Ni/SiO_2$ bimetallic catalysts

Synthesis of $Pd-Ni/SiO_2$ catalysts were performed by selective deposition of Pd on Ni nanoparticles via ALD. Typically, Pd ALD were carried out on the Ni/SiO_2 sample

at 150 °C in a viscous flow reactor (GEMSTAR-6^{TM} Benchtop ALD, Arradiance) using palladium hexafluoroacetylacetate ($Pd(hfac)_2$) and ultrahigh purity H_2 (99.999%) as precursors. Ultrahigh purity N_2 (99.999%) was used as a carrier gas at a flow rate of 200 mL/min. The $Pd(hfac)_2$ precursor container was heated to 65 °C to get a sufficient vapour pressure. The chamber was heated to 150 °C and the inlet manifolds were held at 110 °C to avoid precursor condensation. The timing sequence was 120, 200, 60, and 200 sec for $Pd(hfac)_2$ exposure, N_2 purge, H_2 exposure and N_2 purge, respectively. The obtained Pd-deposited Ni/SiO_2 samples were denoted as *x*Pd-Ni/SiO_2 (*x* represents the number of ALD cycles, and $x = 5, 10, 20$). The Pd loadings were measured as 0.98 wt%, 2.0 wt% and 3.5 wt% for 5Pd-Ni/SiO_2, 10Pd-Ni/SiO_2 and 20Pd-Ni/SiO_2 based on ICP-AES analysis, respectively. As controlled experiments, Pd ALD were applied on the bare SiO_2 support under the same conditions as described above. Such obtained Pd-deposited SiO_2 samples were denoted as *x*Pd/SiO_2 (*x* represents the number of ALD cycles, and $x = 5, 10, 20$). All catalysts were calcined in 10% O_2 in Ar at 150 °C for 2 h, then reduced at 150 °C for 1 h in 10% H_2 in Ar before any reaction tests.

Synthesis of Pd/SiO_2, Pt/SiO_2 catalysts

Following a typical procedure reported previously[2], a Pd/SiO_2 catalyst was synthesized by the wet-impregnation method. Here 0.046 g Palladium acetylacetonate ($Pd(acac)_2$) was dissolved into 50 mL acetylacetone. 400 mg spherical SiO_2 was added into the solution and mixed under vigorous stirring at 25 °C for 24 h. The solvent was slowly evaporated under stirring. The obtained solid was dried at 110 ºC overnight and further calcined at 500 ºC under 10% O_2 in He for 3 h followed by a reduction step at 250 ºC under 10% H_2 in Ar for 2 h to obtain the Pd/SiO_2 catalyst. The loading of Pd was measured as 3.9% based on ICP-AES analysis.

Pt/SiO_2 catalyst was synthesized by ALD method. Typically, Pt ALD were carried out on SiO_2 support (silica gel, Sigma-Aldrich) in the same reactor mentioned above using trimethyl(methylcyclopentadienyl)-platinum(IV) ($MeCpPtMe_3$, 99%) and ultrahigh purity O_2 (99.999%) as precursors at 250 °C. Ultrahigh purity N_2 (99.999%) was used as the carrier gas at a flow rate of 200 mL/min. The Pt precursor was heated

to 65 °C to get a sufficient vapor pressure. The reactor inlets were held at 110 °C to avoid any precursor condensation. The timing sequence was 90, 200, 60, and 200 sec for the $MeCpPtMe_3$ exposure, N_2 purge, O_2 exposure, and N_2 purge, respectively. Two Pt ALD cycles were performed to obtain the Pt/SiO_2 catalyst and the Pt loading was measured as 3.6 wt% base on ICP-AES analysis.

Pt ALD: Synthesis of $1Pt\text{-}Ni/SiO_2$ and $3Pt\text{-}Ni/SiO_2$ bimetallic catalysts

Synthesis of PtNi bimetallic catalysts was performed by selective deposition of Pt on Ni nanoparticles via ALD. Typically, Pt ALD was carried out on the Ni/SiO_2 sample at 150 °C in a home-made viscous flow reactor using trimethyl(methylcyclopentadienyl)-platinum(IV) $MeCpPtMe_3$ and ultrahigh purity O_2 (99.999%) as precursors. Ultrahigh purity N_2 (99.999%) was used as the carrier gas at a flow rate of 200 mL/min. The $MeCpPtMe_3$ precursor container was heated to 65 °C to get a sufficient vapour pressure. The chamber was heated to 150 °C and the inlet manifolds were held at 110 °C to avoid any precursor condensation. The timing sequence was 120, 200, 60, and 200 sec for $MeCpPtMe_3$ exposure, N_2 purge, O_2 exposure and N_2 purge, respectively. After 1 and 3 cycles of Pt ALD, the PtNi bimetallic catalysts were obtained, which were denoted as $1Pt\text{-}Ni/SiO_2$, $3Pt\text{-}Ni/SiO_2$, respectively. The obtained catalysts were calcined in 10% O_2 in Ar at 150 °C for 2 h, then reduced at 150 °C for 1 h in 10% H_2 in Ar before reaction test.

Detailed metal loadings of various Pd, Pt and bimetallic PdNi catalysts can be seen in Supplementary Table 1. All catalysts were calcined in 10% O_2 in Ar at 150 °C for 2 h, then reduced at 150 °C for 1 h in 10% H_2 in Ar before any reaction tests.

Catalysts characterization

Materials morphology and compositions characterization

TEM measurements were performed on a JEM-2100F instrument (University of Science and Technology of China). HAADF-STEM images and corresponding EDS elemental mapping and line-scan analysis results were collected on an aberration-corrected JEM-ARM 200F instrument (University of Science and Technology of

China). The elemental analysis of metal contents in various catalysts were analyzed by ICP-AES (Optima 7300DV); therein all samples were dissolved in hot aqua regia.

In-situ DRIFTS CO chemisorption

The diffuse reflectance infrared Fourier transform spectroscopy (DRIFTS) CO chemisorption measurements were performed on a Nicolet iS10 spectrometer equipped with a mercury cadmium telluride (MCT) detector and a low-temperature reaction cell (Praying Mantis Harrick). After loading a sample into the cell, the sample was first calcined in 10% O_2 in Ar at 150 °C for 1 h followed by reduction in 10% H_2 in Ar at 150 °C for another 1 h. After cooling the sample to room temperature under a continuous flow of Ar, a background spectrum was collected. Subsequently, the sample was exposed to 10% CO in Ar at a flow rate of 20 mL/min for about 30 min until saturation. Next, the sample was purged with Ar at a flow rate of 20 mL/min for 30 min to remove the gas phase CO and then purged with 10% O_2 in Ar for another 30 min to remove the CO adsorbed on metallic Ni surface, by oxidizing the Ni surface, so that the CO chemisorbed on Pd can well distinguished. The DRIFT spectrum was collected with 256 scans at a resolution of 4 cm^{-1}.

In-situ XPS measurements

In-situ X-ray photoemission spectroscopy (XPS) measurements were conducted at the BL10B beamline Photoemission End-station at the National Synchrotron Radiation Laboratory (NSRL) in Hefei, China. Briefly, the beamline is connected to a bending magnet and covers photon energies from 100 to 1000 eV. The end-station consists of four chambers, i.e., an analysis chamber, a preparation chamber, a quick sample load-lock chamber and a high pressure reactor. The analysis chamber, with a base pressure of $<5\times10^{-10}$ torr, is connected to the beamline and equipped with a VG Scienta R3000 electron energy analyzer and a twin anode X-ray source. The high pressure reactor houses a reaction cell where the samples can be treated with different gases up to 20 bar and simultaneously heated up to 650 °C. After sample treatment, the reactor was pumped down to high vacuum ($<10^{-8}$ torr) for sample transfer. In the current work, the

samples were first treated with the flowing 10% O_2 in Ar at 150 °C for 1 h followed by reduction in 10% H_2 in Ar (20 mL/min) at 150 °C for 0.5 h at the ambient pressure in the high pressure reactor. Next, the samples were transferred to the analysis chamber for XPS measurements in the Pd 3d region without exposing to air.

In situ XAFS spectroscopy

In situ X-ray absorption fine structure (XAFS) measurements at Pd *K*-edge (24350 eV) were performed with the Si(311) monochromator at the BL14W1 beamline of the Shanghai Synchrotron Radiation Facility (SSRF), China. The storage ring of SSRF worked at 3.5 GeV with a maximum current of 210 mA. The XAFS spectrum at Pd *K*-edge of 5Pd-Ni/SiO_2 sample was recorded in the fluorescence mode, while the XAFS spectra of 10Pd-Ni/SiO_2 and 20Pd-Ni/SiO_2 were recorded in the transmission mode, when the loadings of Pd in these samples were taken into account. The energy was calibrated accordingly to the absorption edge of Pd foil. All catalysts were first pressed into sample pellets and were then loaded into a home-made quartz reaction cell, where Kapton foil was used as the X-ray window material. This quartz reaction cell can be heated to 500 °C with external heating. A K-type thermocouple, protected by a closed-end quartz tube, was located near the sample pellet to measure the sample temperature. After loading into the reaction cell, each sample was first reduced in 10% H_2 in He at 150 °C for 30 min (20 mL/min); Next, the sample was purged in ultrahigh purity He at 150 °C for 15 min and cooling to room temperature, then a XAFS spectrum was recorded.

Catalytic performance tests

Tests of catalytic hydrogenation of nitriles

The catalytic reaction of hydrogenation of nitriles was conducted in a 100 mL stainless-steel autoclave (NS100-SV, Anhui Kemi Machinery Technology Co., Ltd) with Teflon inlet. In a typical test, 0.5 g benzonitrile, 30 mg catalysts and 60 ml ethanol were co-added into the autoclave. After three cycles flush with ultrahigh purity He, the autoclave was pressurized with 0.6 MPa hydrogen. The reaction proceeded under

magnetic stirring with a rate of 1200 r.p.m. at 80 °C. During the reaction, the liquid sample mixture was take out through a sampling valve and analyzed by gas chromatography (Shimadzu GC-2014, equipped with an Rtx-1 capillary column and auto-injector). Dodecane was used as internal standard. The TOFs were calculated using the following equation (1):

$$TOFs = \frac{moles\ of\ nitrile\ converted}{moles\ of\ total\ Pd\ (Pt)\ atoms\ \times reaction\ time} \qquad (1)$$

Here, the TOFs were evaluated after proceeding the reaction for 60 min unless otherwise noted.

To evaluate the stability of the catalysts, 1 g benzonitrile, 100 mg catalysts and 60 ml ethanol were co-added into the autoclave. The autoclave was flushed with He for three times and then was pressurized with 0.6 MPa hydrogen. After catalytic reaction proceeded at 80 °C for 2 h, the autoclave was cooled to room temperature and the hydrogen was released, then the products were analyzed. The catalysts were collected by centrifugation and washed three times with ethanol carefully, followed by re-adding to the Teflon inlet for the next round without any pre-treatment.

Tests of catalytic hydrogenolysis of benzylamines

The tests of hydrogenolysis reaction followed a similar procedures with ones used in hydrogenation nitriles, except for changing the substrate from nitriles to benzylamine. The TOFs were calculated using the following equation (2):

$$TOFs = \frac{moles\ of\ amines\ converted}{moles\ of\ total\ Pd\ (Pt)\ atoms\ \times reaction\ time} \qquad (2)$$

Here, the TOFs were evaluated after proceeding the reaction for 60 min unless otherwise noted.

Theoretical calculations

Density functional theory (DFT) with the PBE (Perdew-Burke-Erzenhorf)[3] functional as implemented in the Vienna ab initio Simulation Package (VASP)[4,5] was employed. The projector augmented wave method[6,7] was used to describe the interaction between the ions and the electrons. A cutoff energy of 450 eV was used for

the plane-wave basis set. The van der Waals interactions described by the empirical correction in Grimme scheme (vdW-D3)[8] was also adopted in this work. The geometry optimization conversions with forces on atoms and energy differences were smaller than 0.02 eV/Å and 10^{-5} eV, respectively. Pd(111), Pt(111), and Ni(111), were modeled by four-layer slabs with (4×4) unit cells, and the top two layers were fully relaxed as well as the bottom two layers were fixed at their bulk positions. The vacuum slab was set up to 15 Å along the z direction. The model with one surface atom of Ni(111) replace by a Pd atom (denoted as $Pd_1Ni(111)$) represents the Pd_1Ni SASA catalyst. The Brillouin zone was sampled with a grid of 3 × 3 × 1 mesh points according to the Monkhorst-Pack procedure.[9] The nudged elastic band (NEB)[10] approach was employed to locate the transition state (TS) between a reactant (initial state: IS) and its product (final state: FS).

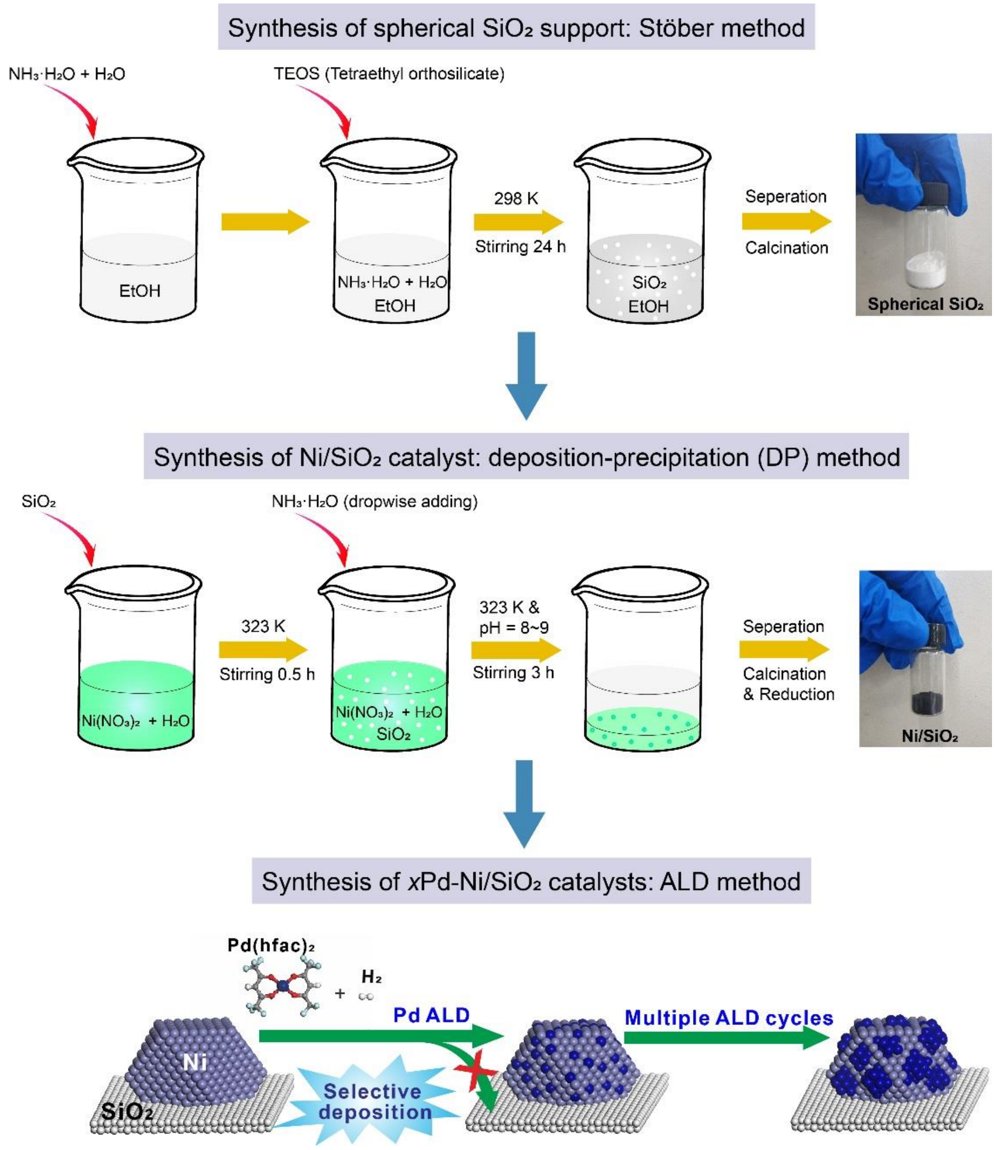

Supplementary Figure 1 | Schematic illustration of synthesis of *x*Pd-Ni/SiO_2 bimetallic catalysts by combing wet-chemistry with selective Pd ALD.

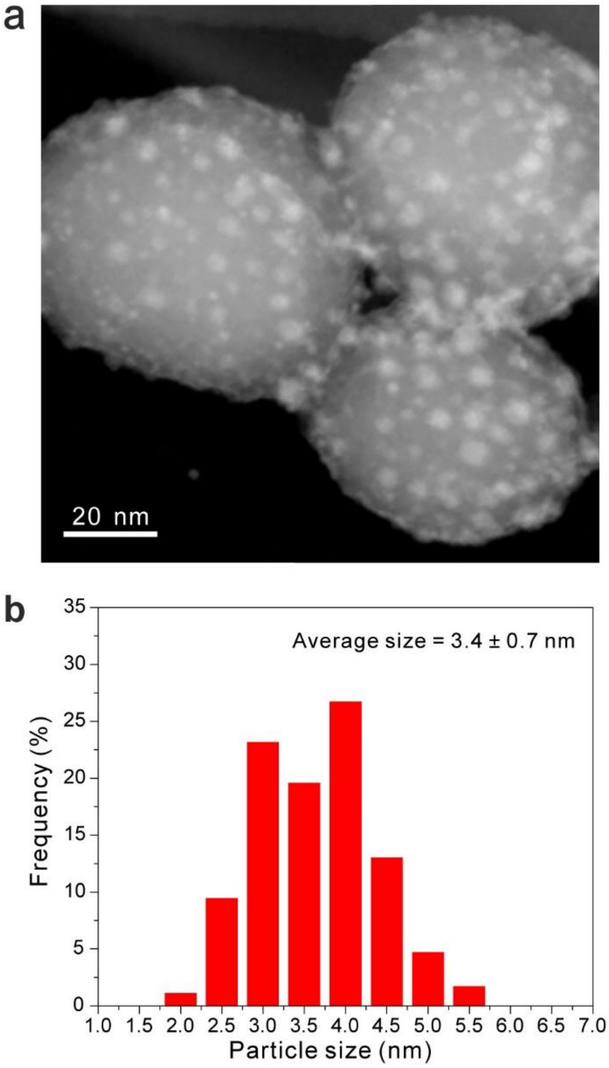

Supplementary Figure 2 | Morphology of the 3.4 nm Ni/SiO_2 catalyst. a, A representative STEM image. **b,** The corresponding Ni particle size distribution.

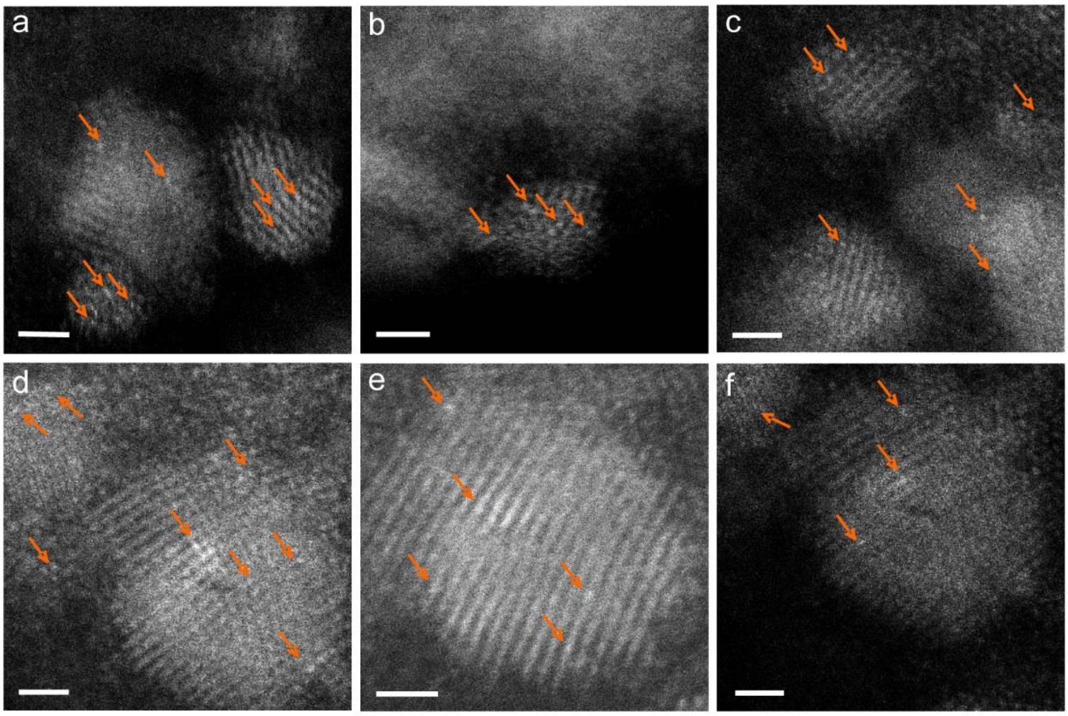

Supplementary Figure 3 | Representative HAADF-STEM images of 5Pd-Ni/SiO_2 at different locations (a-f). Isolated Pd single atoms on partially-oxidized Ni NPs are highlighted by brown arrows. The scale bar is 2 nm in all images.

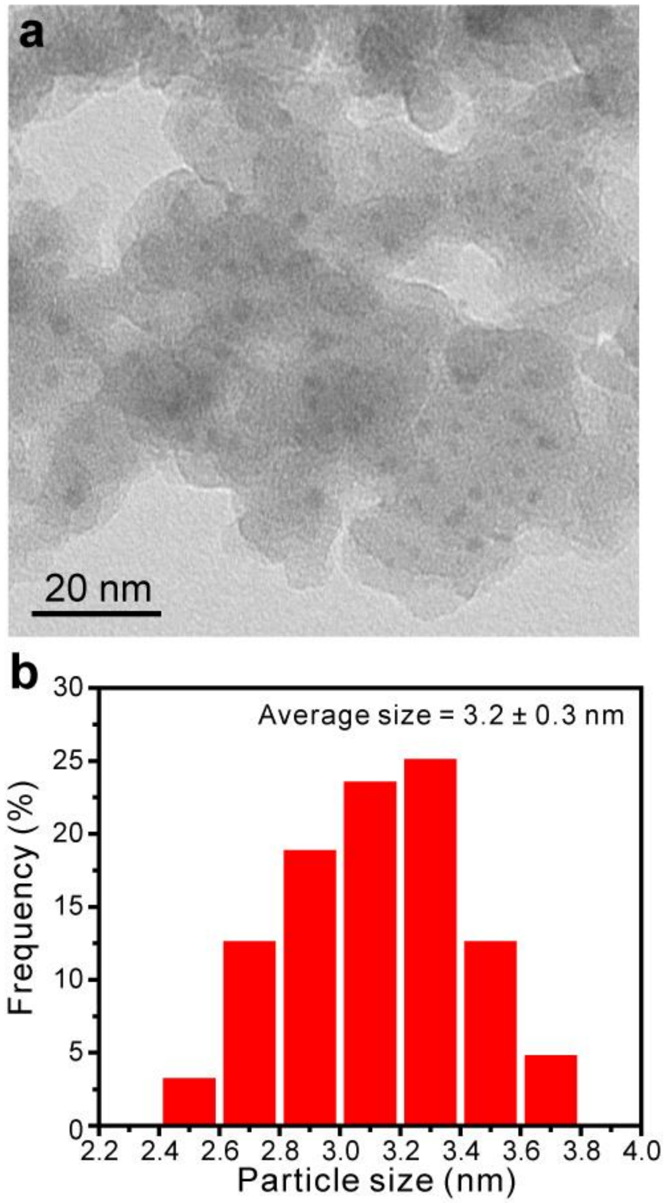

Supplementary Figure 4 | Morphology of the 3.2 nm Pd/SiO_2 catalyst. a, A representative TEM image. **b,** The corresponding Pd particle size distribution.

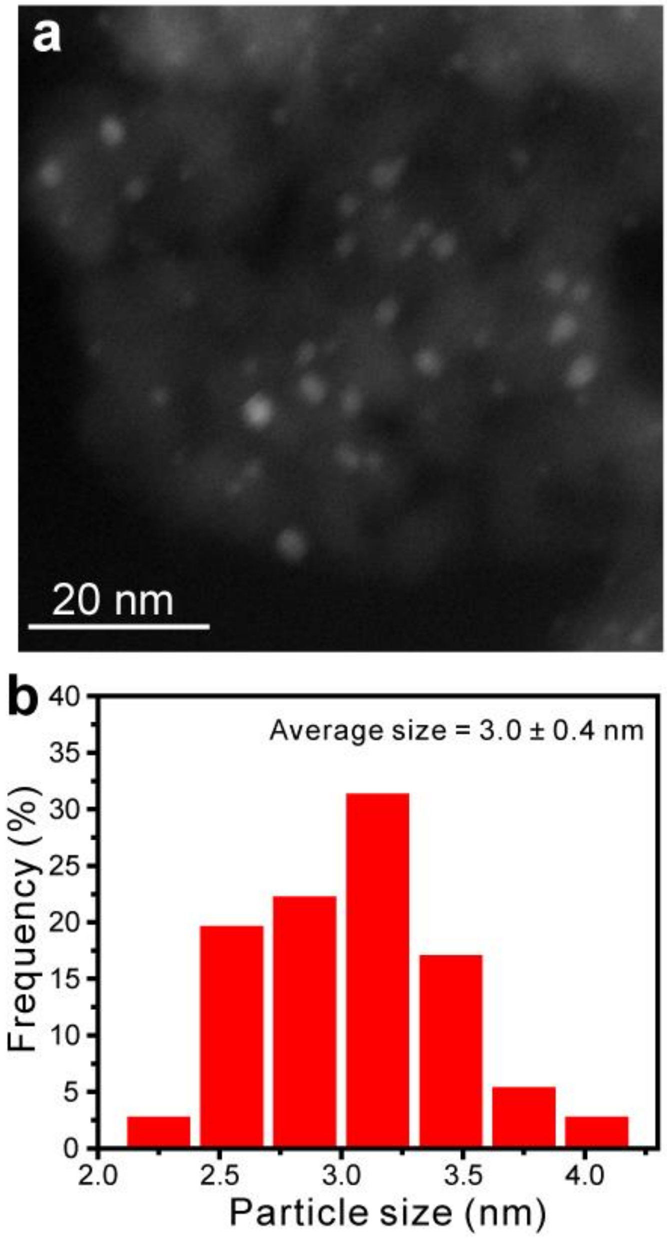

Supplementary Figure 5 | Morphology of the 3.0 nm Pt/SiO_2 catalyst. a, A representative STEM image. **b,** The corresponding Pt particle size distribution.

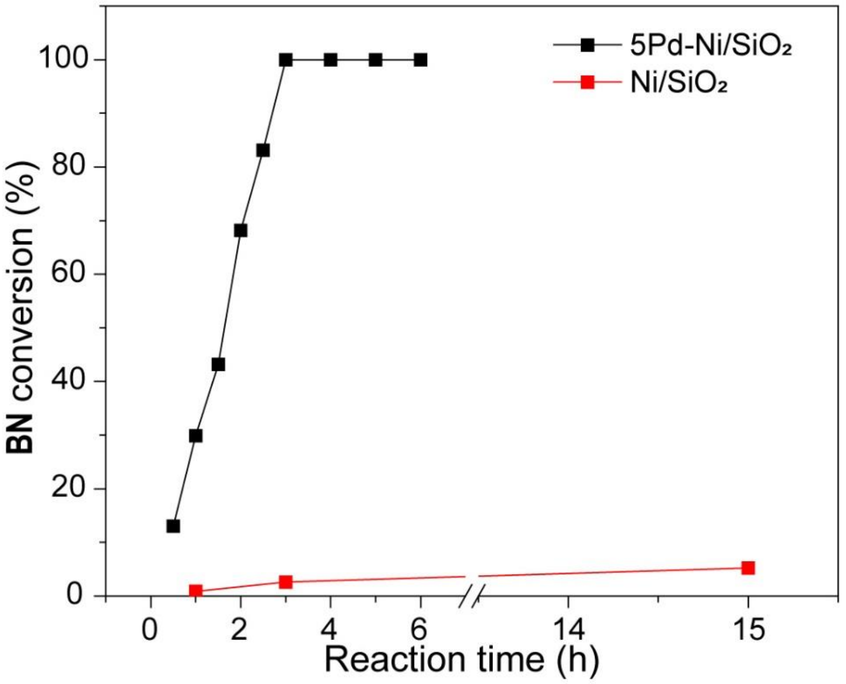

Supplementary Figure 6 | Catalytic performance of 3.4 nm Ni/SiO_2 catalyst in hydrogenation of BN. Reaction conditions: Solvent, ethanol, 60 mL; BN, 0.5 g; catalyst, 30 mg; H_2 pressure, 0.6 MPa; temperature, 80 ºC.

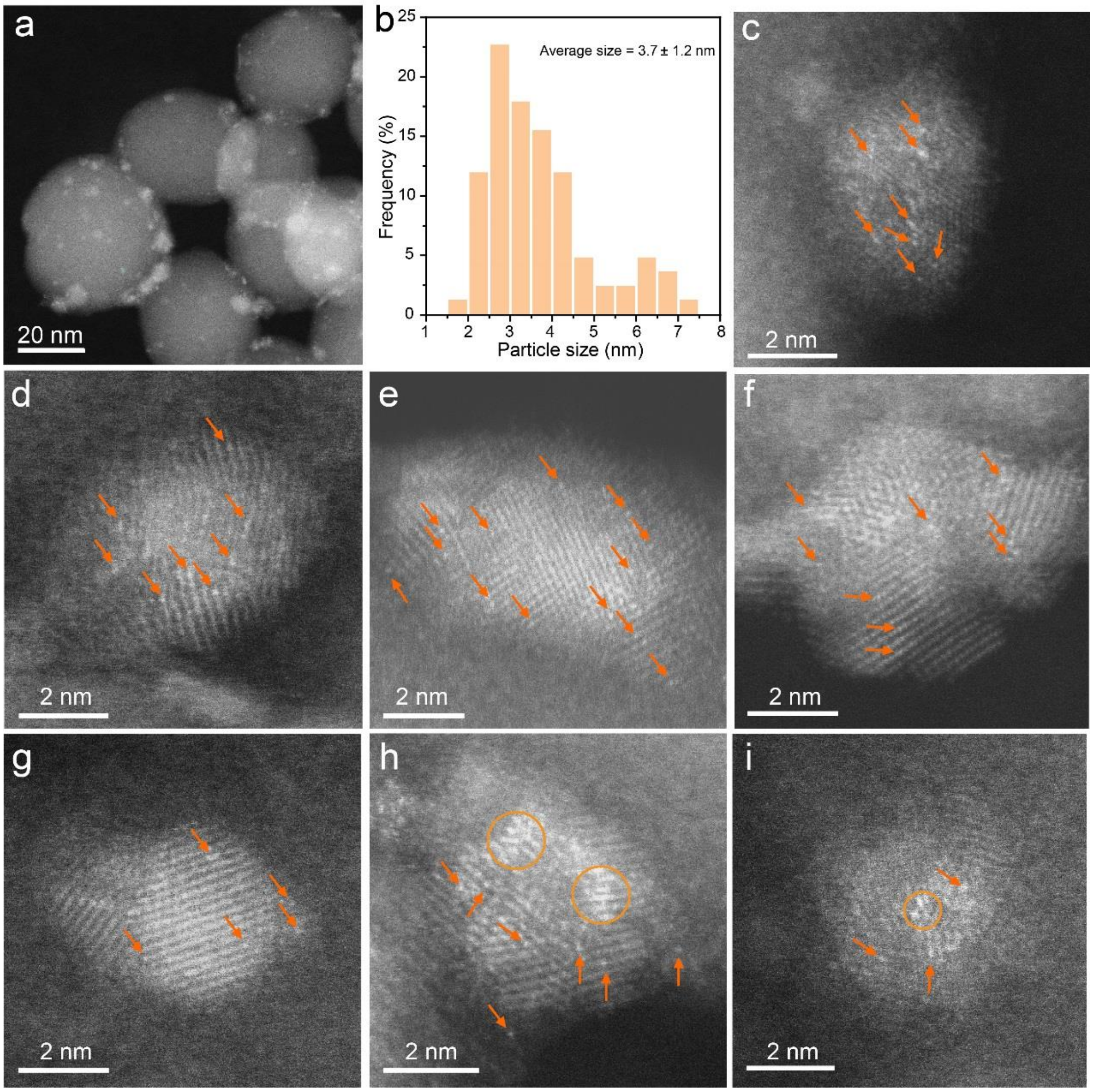

Supplementary Figure 7 | Representative HAADF-STEM images of 5Pd-Ni/SiO_2 after 8-cycle-run recyclability test. **a**, A low-magnification STEM image. **b**, The corresponding particle size distribution. **c-i**, High-magnification STEM images at different locations. Isolated Pd single atoms on partially-oxidized Ni NPs are highlighted by brown arrows. In addition, small Pd ensembles (highlighted by brown circles in **h** and **i**) were also observed which is consistent with the DRIFTS CO chemisorption and XAFS results.

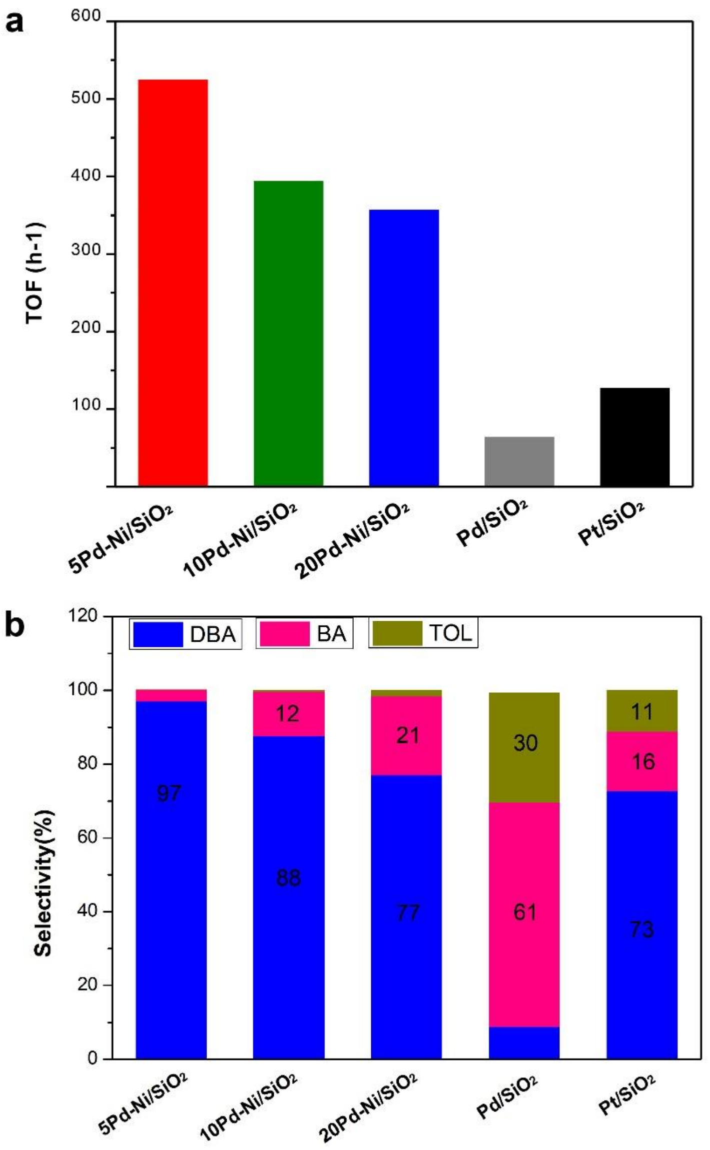

Supplementary Figure 8 | Catalytic performance of Pd/SiO_2, Pt/SiO_2 and *x*Pd-Ni/SiO_2 (*x* = 5, 10 and 20) samples in hydrogenation of BN. a, TOFs. **b,** The corresponding selectivity to different products at 100% BN conversion**.** Reaction conditions: Solvent, ethanol, 60 mL; BN, 0.5 g; catalyst, 30 mg; H_2 pressure, 0.6 MPa; temperature, 80 ºC.

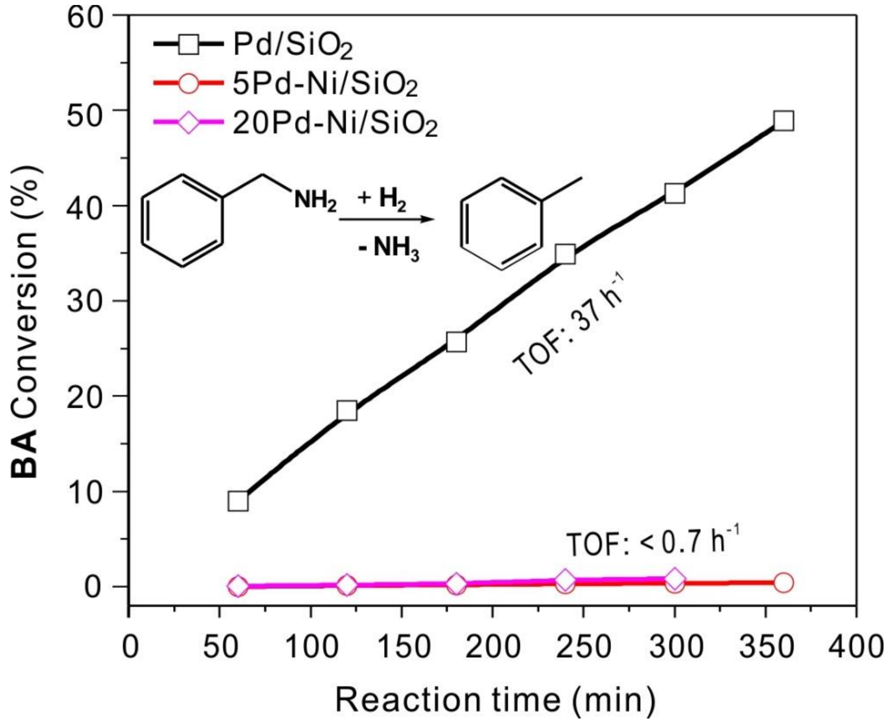

Supplementary Figure 9 | Time profiles of hydrogenolysis of BA over Pd/SiO$_2$, 5Pd-Ni/SiO$_2$ and 20Pd-Ni/SiO$_2$. Reaction conditions: Solvent, ethanol, 60 mL; BA**,** 0.5 g; catalyst, 30 mg; H_2 pressure, 0.6 MPa; temperature, 80 ºC.

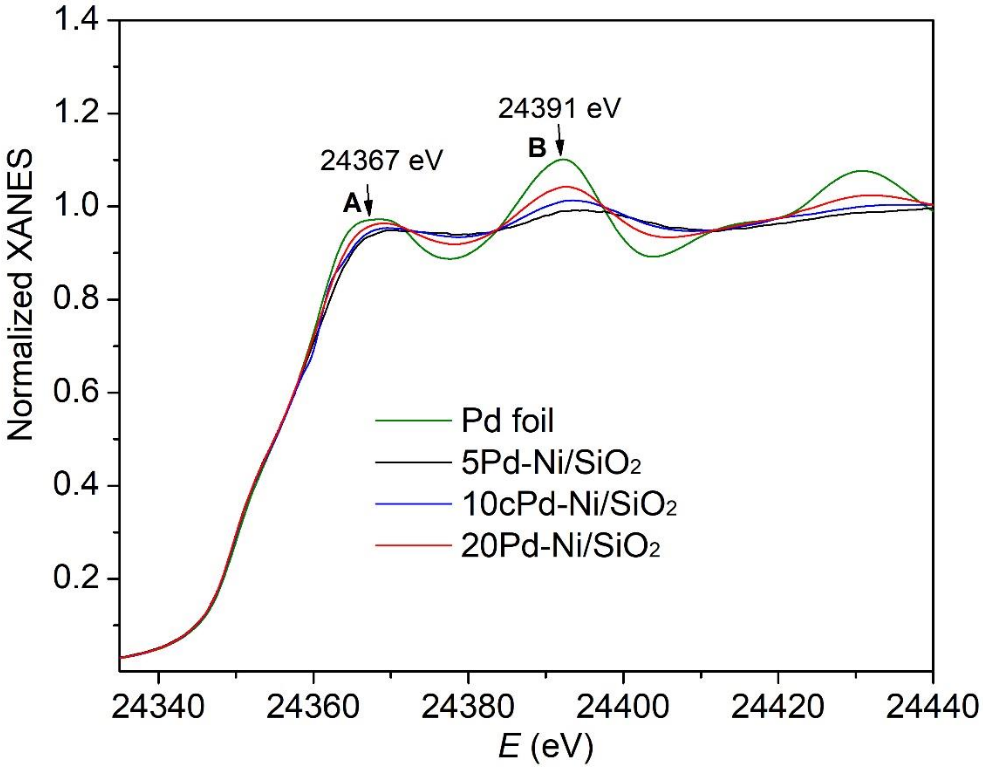

Supplementary Figure 10 | In situ XANES spectra of the *x*Pd-Ni/SiO_2 samples (*x* = 5, 10 and 20) and a Pd foil reference at the Pd *K*-edge.

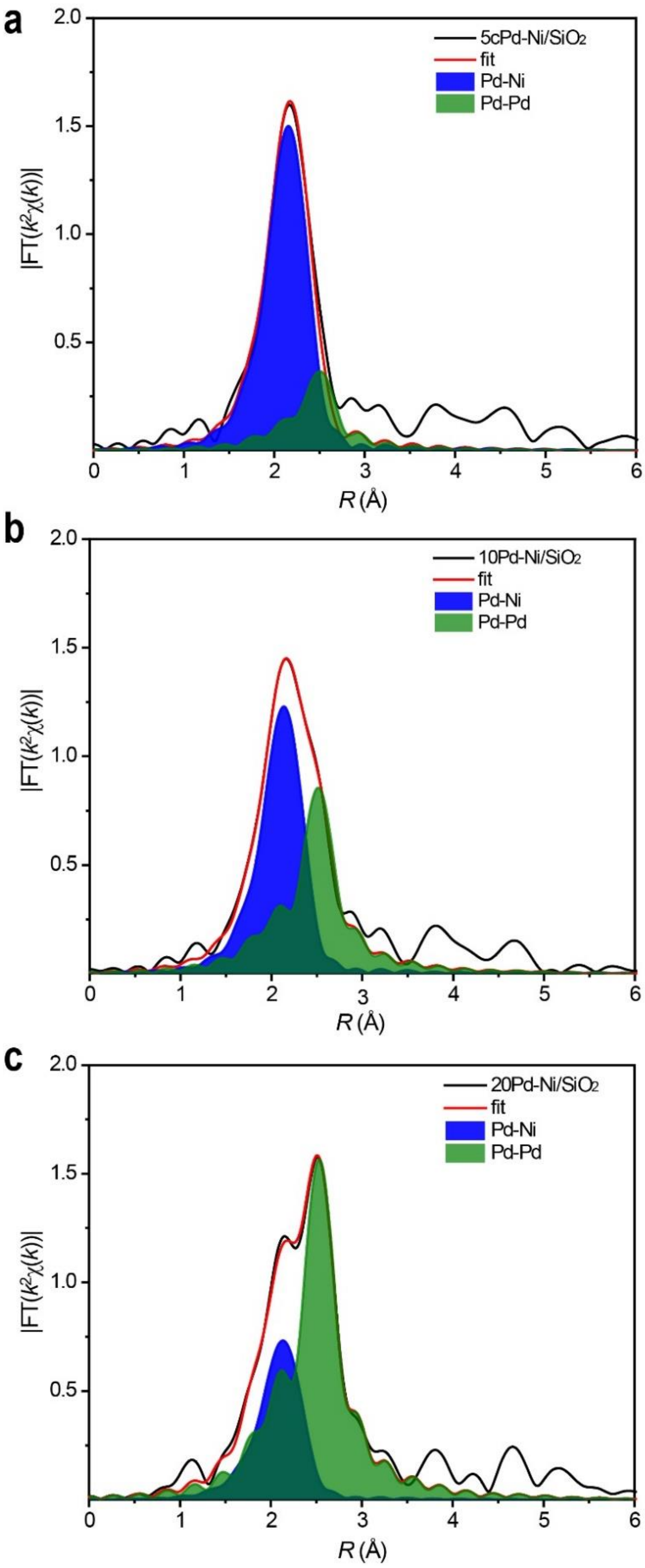

Supplementary Figure 11 | The fitting curves of k^2-weighted EXAFS spectra of the *x*Pd-Ni/SiO_2 samples (*x* = 5, 10 and 20). a, 5Pd-Ni/SiO_2. **b**, 10Pd-Ni/SiO_2. **c**, 20Pd-Ni/SiO_2.

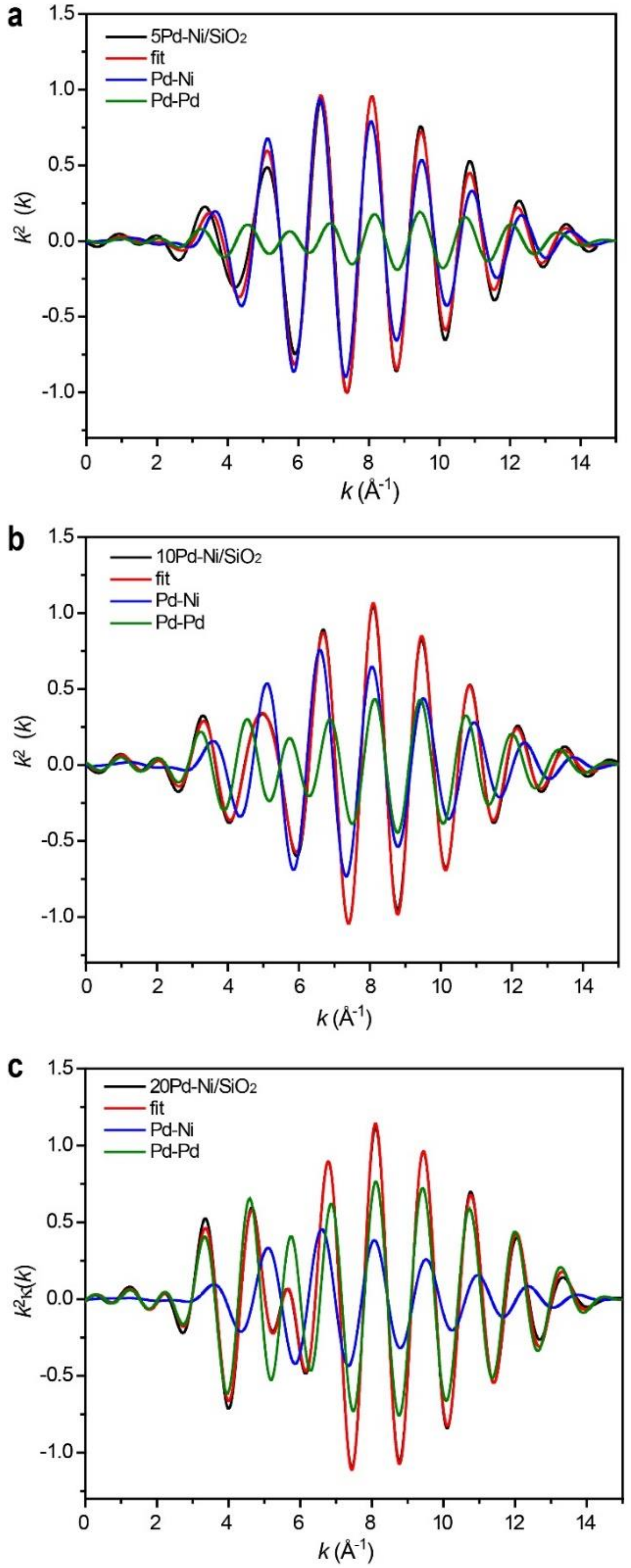

Supplementary Figure 12 | The fitting curves of $k^2\chi(k)$ oscillations of the *x*Pd-Ni/SiO$_2$ samples (*x* = 5, 10 and 20). a, 5Pd-Ni/SiO$_2$. **b**, 10Pd-Ni/SiO$_2$. **c**, 20Pd-Ni/SiO$_2$.

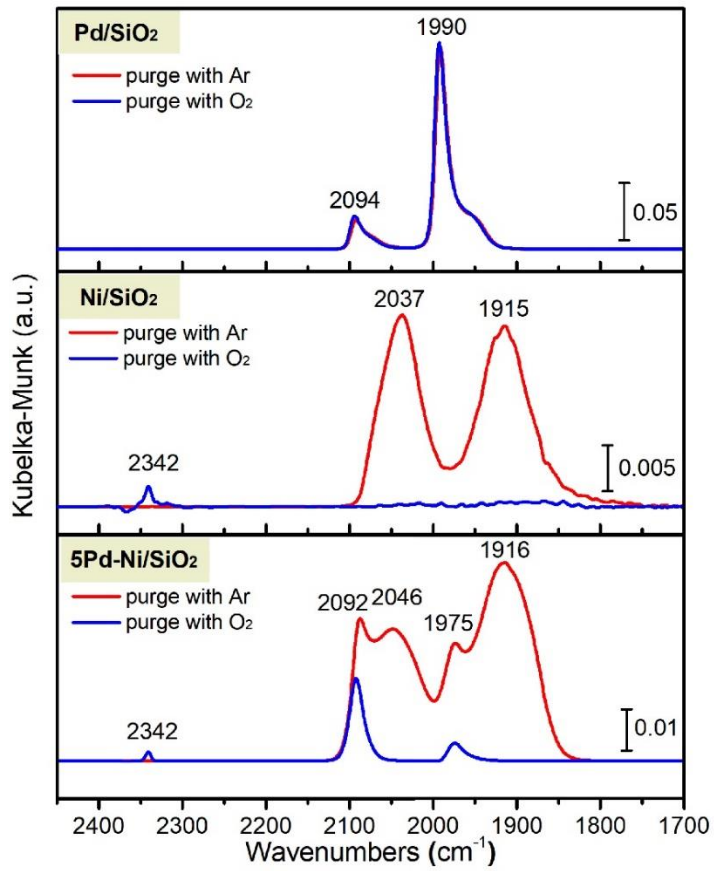

Supplementary Figure 13 | DRIFTS CO chemisorption of the Pd/SiO_2, Ni/SiO_2 and 5Pd-Ni/SiO_2 samples at the CO saturation coverage.

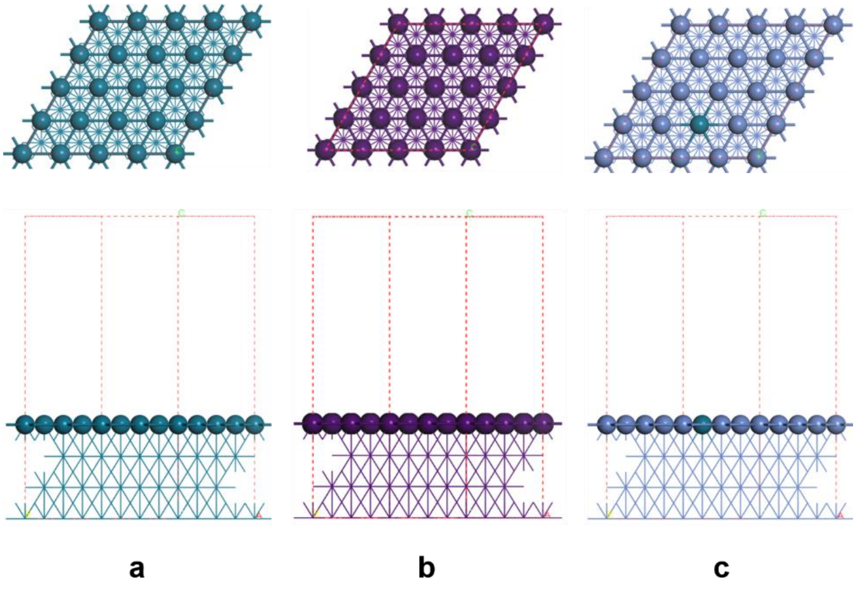

Supplementary Figure 14 | Four-layer slabs models with (4×4) unit cells with top and side views. a, Pd(111). **b**, Pt(111). **c**, Pd_1@Ni(111). The Pd, Pt, Ni atoms are shown in green, violet and light blue, respectively.

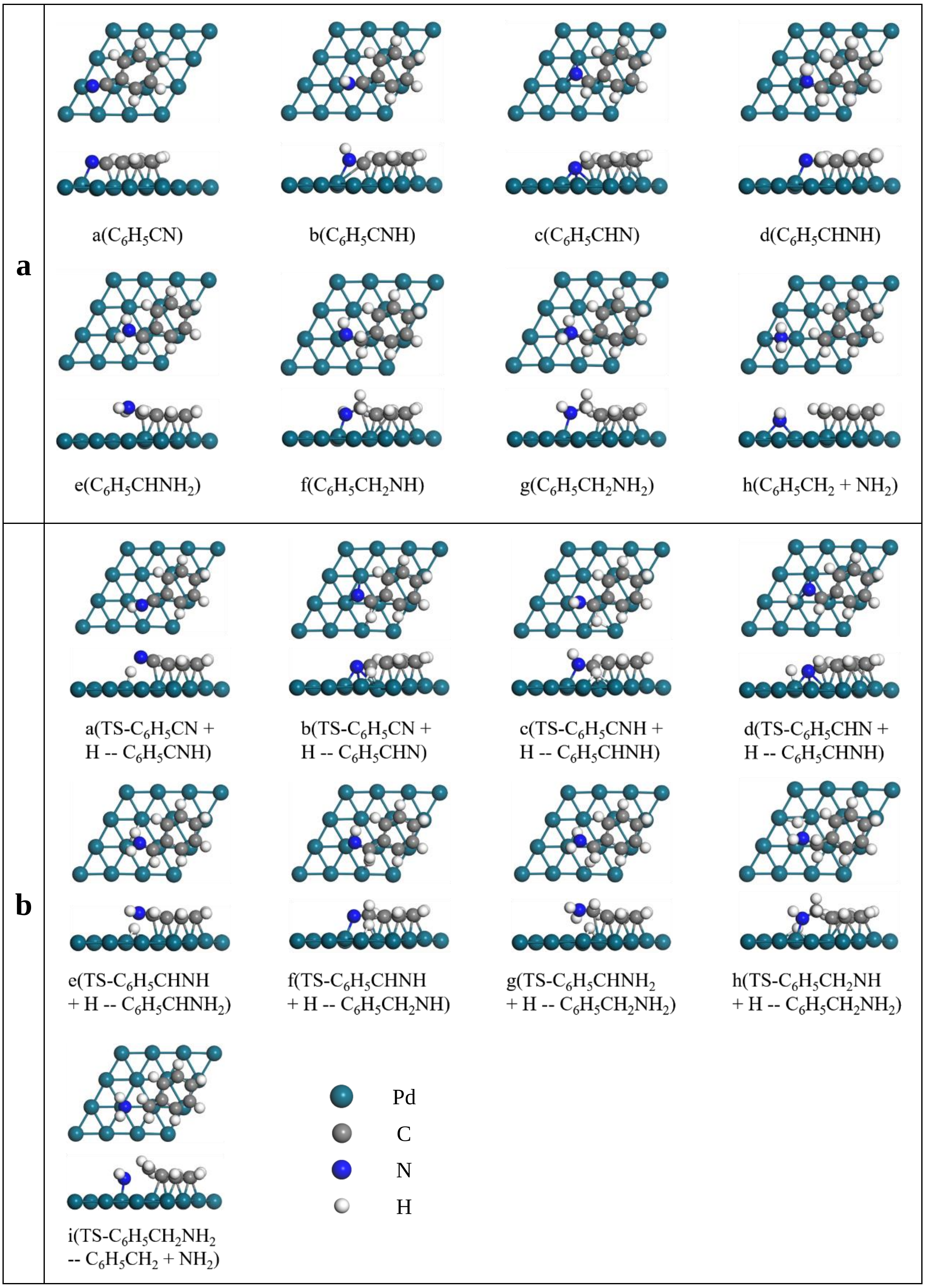

Supplementary Figure 15 | Optimized geometries of BN, its derived key intermediates and transition states on Pd(111). a, The top and side views of optimized geometries of benzonitrile and its derived key intermediates. **b**, The transition states of elementary steps involved in benzonitrile hydrogenation. The legends in **b** also applies to those in **a**.

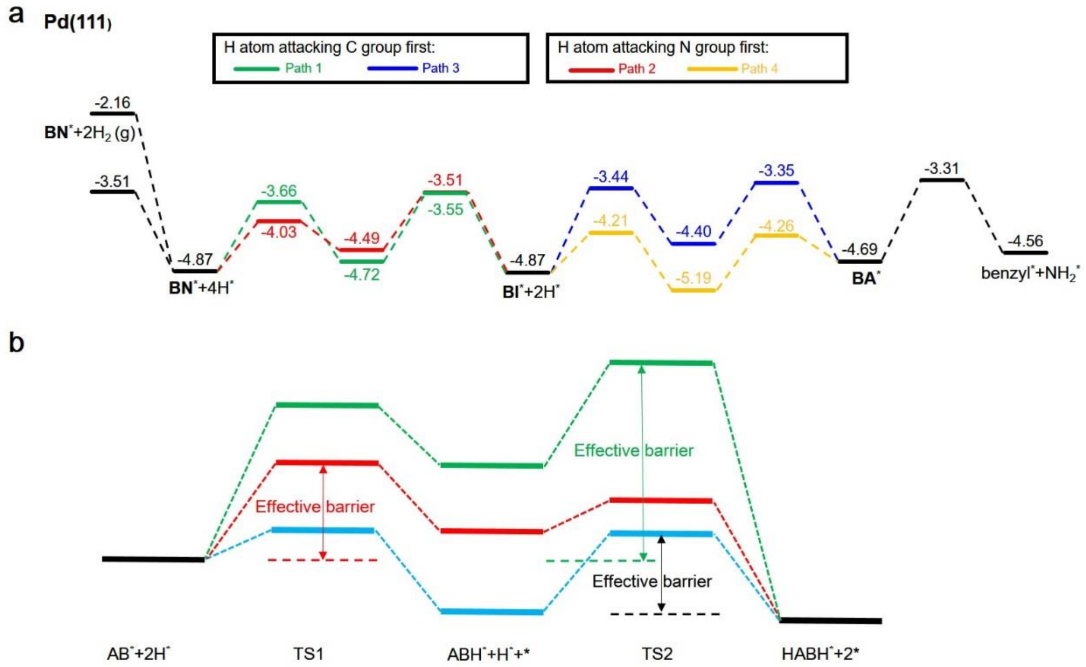

Supplementary Figure 16 | Energy profiles of the reaction paths in BN hydrogenation and the sequential BA hydrogenolysis on Pd(111). a, Energy profiles**. b,** An illustration of the definition of effective energy barriers.

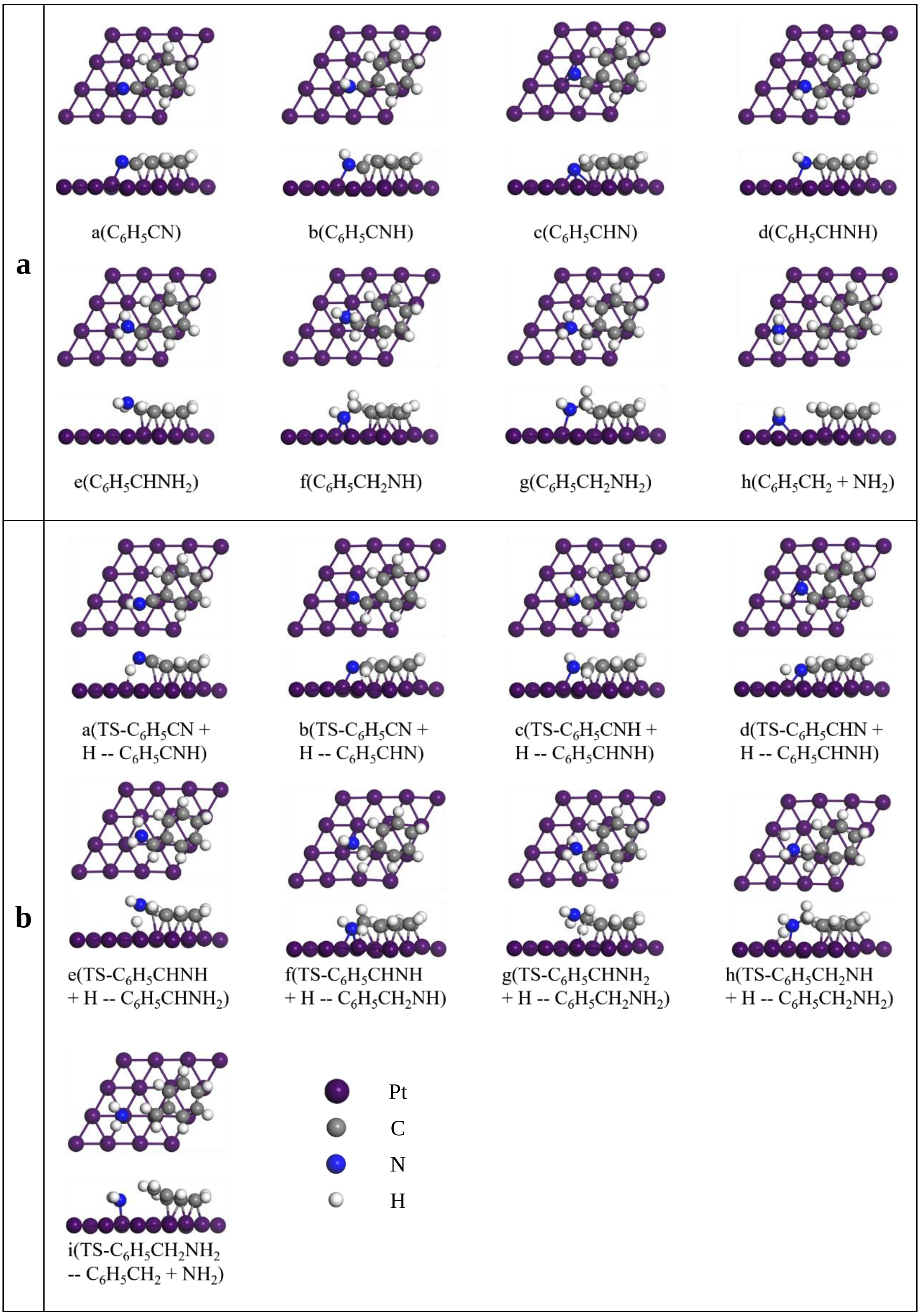

Supplementary Figure 17 | Optimized geometries of BN, its derived key intermediates and transition states on Pt(111). a, The top and side views of optimized geometries of benzonitrile and its derived key intermediates. **b**, The transition states of elementary steps involved in benzonitrile hydrogenation. The legends in **b** also applies to those in **a**.

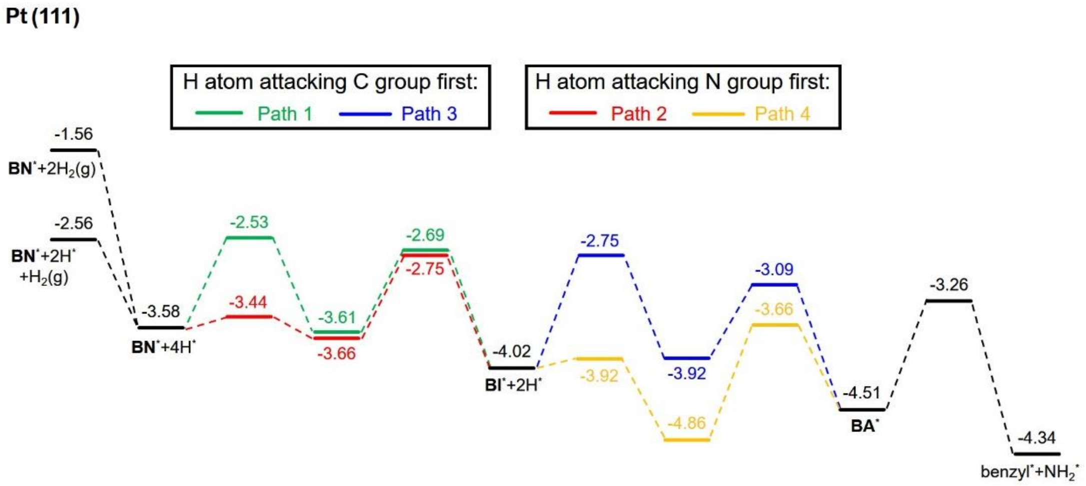

Supplementary Figure 18 | Energy profiles of the reaction paths in BN hydrogenation and the sequential BA hydrogenolysis on Pt(111).

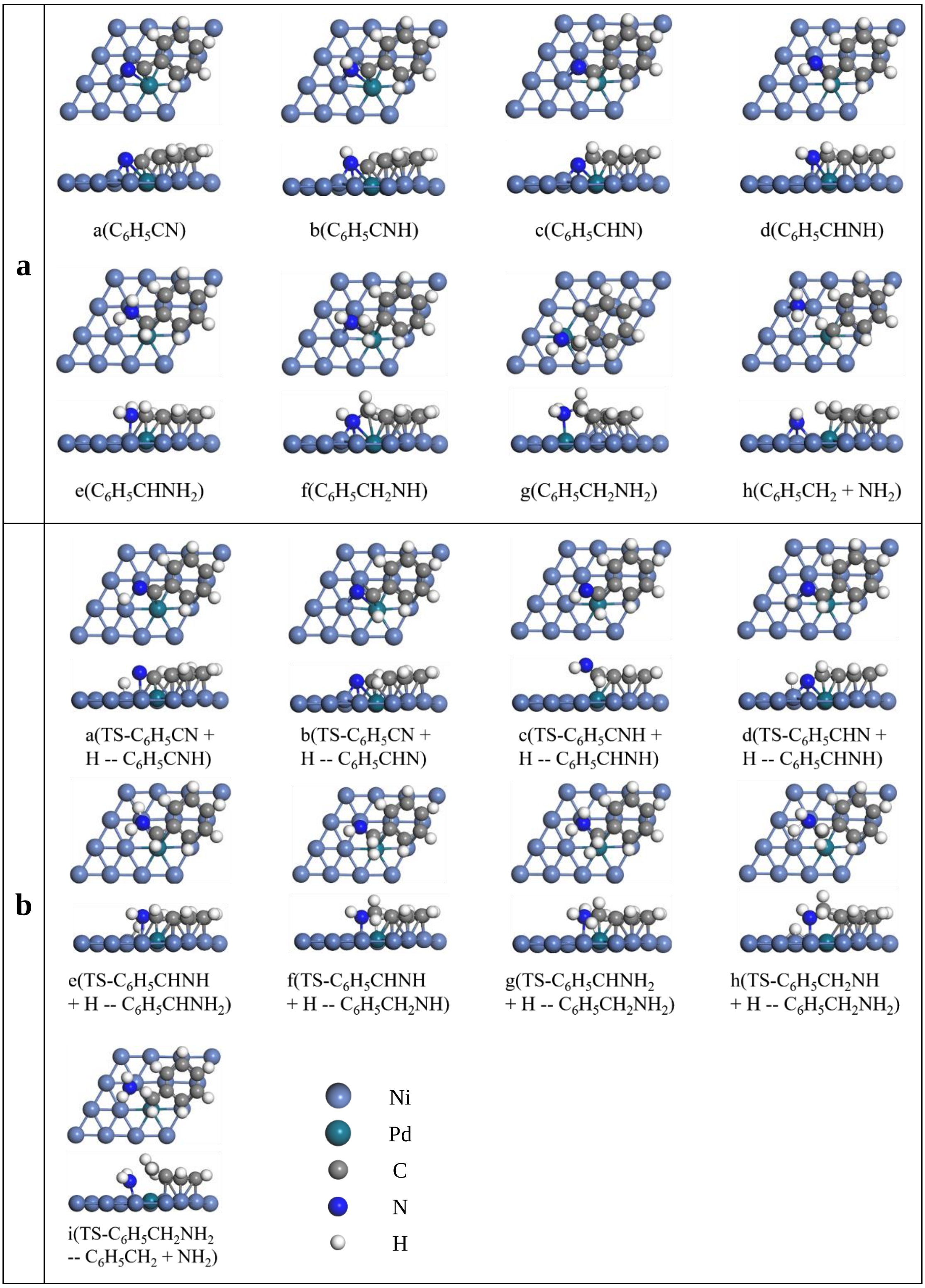

Supplementary Figure 19 | Optimized geometries of BN, its derived key intermediates and transition states on Pd1@Ni(111). a, The top and side views of optimized geometries of benzonitrile and its derived key intermediates. **b,** The transition states of elementary steps involved in benzonitrile hydrogenation. The legends in **b** also apply to **a**.

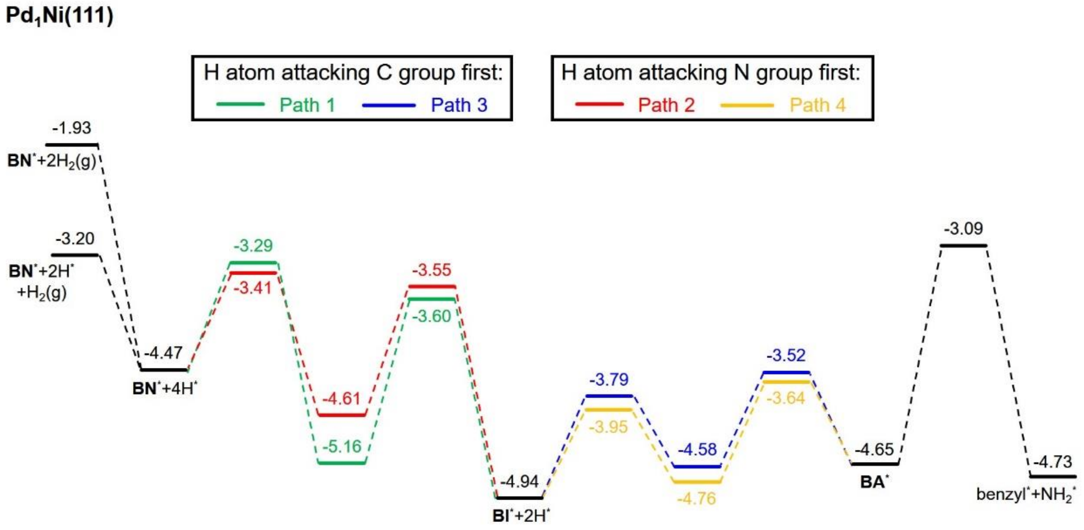

Supplementary Figure 20 | Energy profiles of the reaction paths in BN hydrogenation and sequential BA hydrogenolysis on Pd_1@Ni(111).

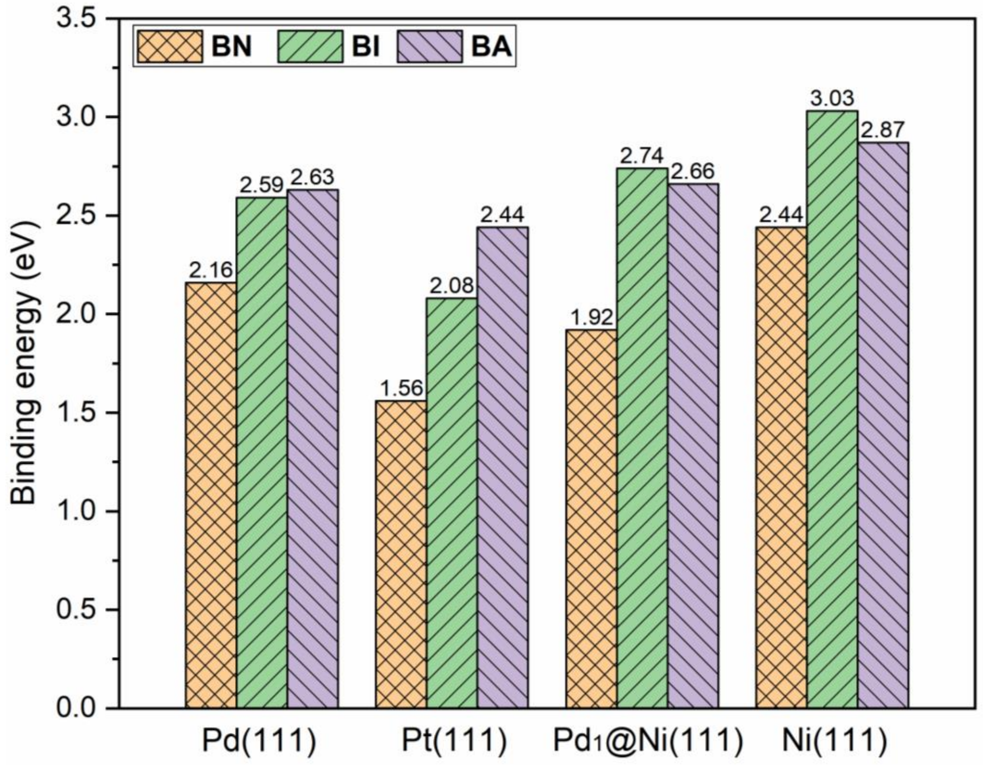

Supplementary Figure 21 | Adsorption energies of the BN, BA and the BI intermediate on Pd(111), Pt(111), Pd_1@Ni(111) and Ni(111) surfaces.

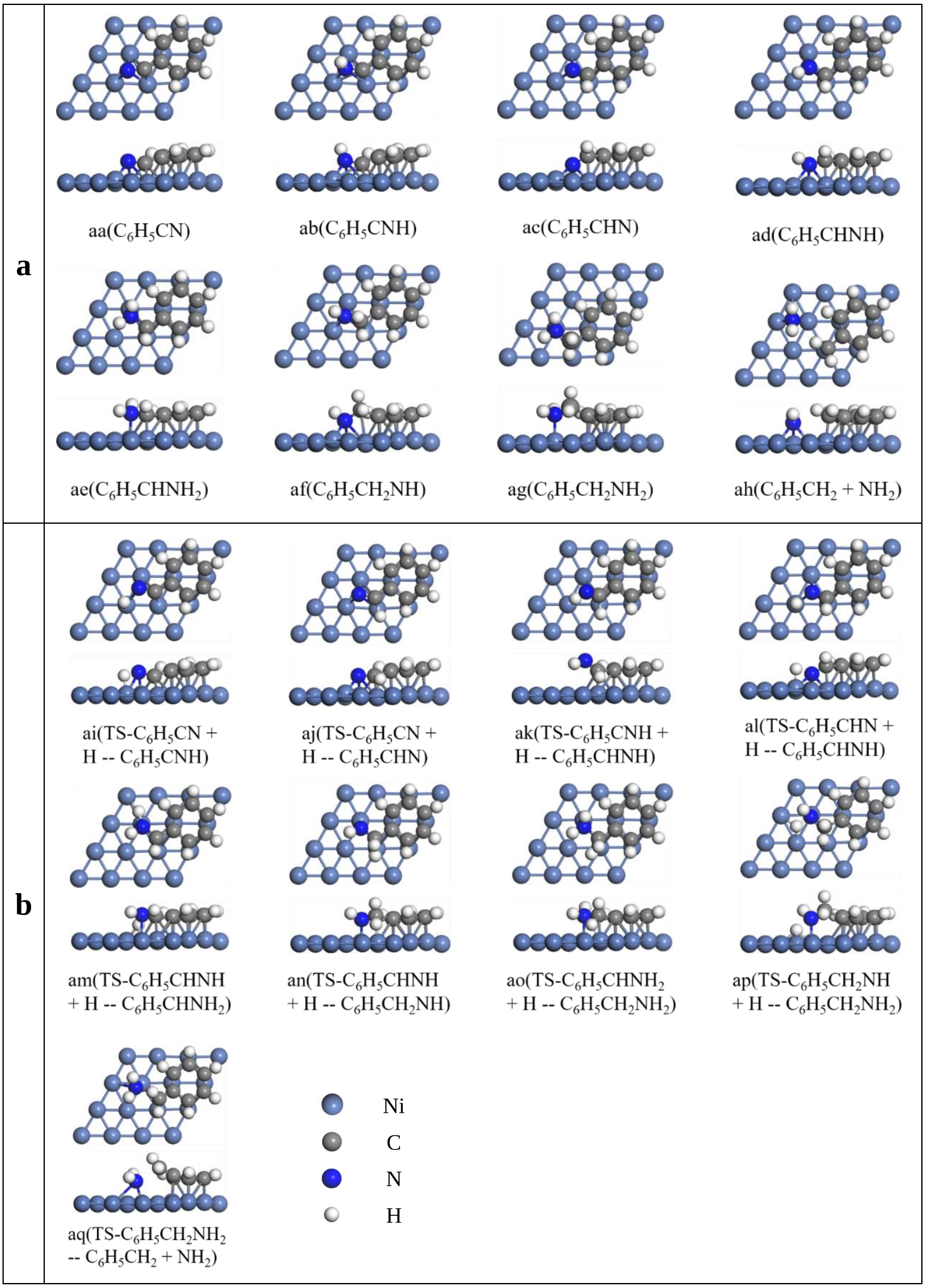

Supplementary Figure 22 | Optimized geometries of BN, its derived key intermediates and transition states on Ni(111). a, The top and side views of optimized geometries of benzonitrile and its derived key intermediates. **b**, The transition states of elementary steps involved in benzonitrile hydrogenation. The legends in **b** also applies to those in **a**.

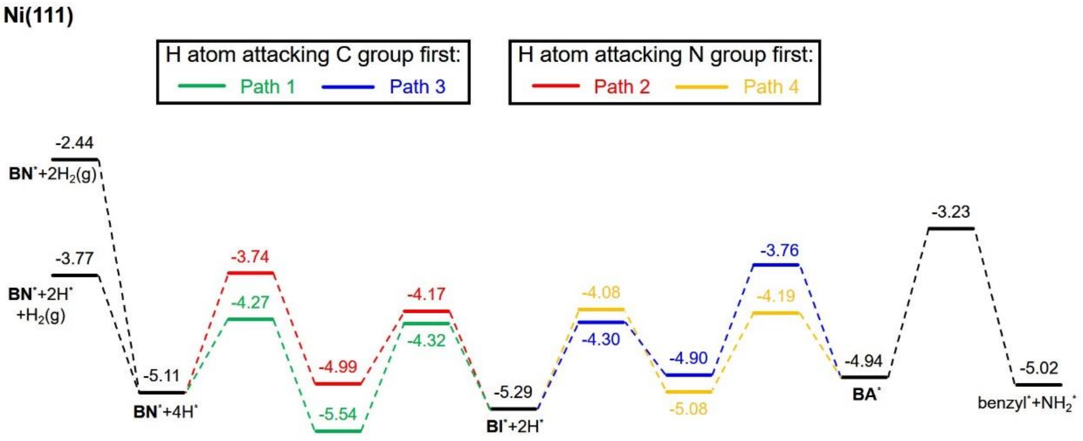

Supplementary Figure 23 | Energy profiles of the reaction paths in BN hydrogenation and sequential BA hydrogenolysis on Ni(111).

Supplementary Table 1 | Metal loadings of various catalysts.

Sample	Metal loadings (wt%)		
	**Ni**	**Pd**	**Pt**
Ni/SiO_2	19	-	-
$5Pd\text{-}Ni/SiO_2$	19	0.98	-
$10Pd\text{-}Ni/SiO_2$	19	2.0	-
$20Pd\text{-}Ni/SiO_2$	19	3.5	-
Pd/SiO_2	-	3.9	-
Pt/SiO_2	-	-	4.0
$1Pt\text{-}Ni/SiO_2$	19	-	0.4
$3Pd\text{-}Ni/SiO_2$	19	-	0.9

Supplementary Table 2 | A comparison of catalysts performance in terms of selectivity and activity in hydrogenation of BN.

Catalysts		T (°C), H_2 pressure (MPa)	Reaction time (h)	Conversion (%)	Selectivity (%)			TOF † (h^{-1})	Notes	
					BA	DBA	TOL		Additional information	References
Pd-based	**5Pd-Ni/SiO$_2$**	**80, 0.6**	1	30	**3**	**97**	**N.D.**	**515**		This work
		**80, 0.6**	3	100	**3.5**	**96.5**	**N.D.**			
	Pd/γ-Al$_2$O$_3$	100, 2.1	0.3	20	84	6	10	540 (*1320)	Adding Ir into Pd prompt the formation of DBA	Ref [11]
	Pd$_{80}$-Ir$_{20}$/γ-Al$_2$O$_3$	100, 2.1	n.a.	20	8	84	8	*120		
	Pd$_{50}$-Ir$_{50}$/γ-Al$_2$O$_3$	100, 2.1	0.25	20	66	16	18	650 (*880)		
	Pd$_{20}$-Ir$_{80}$/γ-Al$_2$O$_3$	100, 2.1	n.a.	20	15	71	14	*42		
	Pd/Al$_2$O$_3$	70, 1.5	WHSV = 0.5 h^{-1}	96.7	88.9	0	9.3	100	Trickle-bed reactor; WHSV = 0.2 - 0.5 g g_{cat} h^{-1}	Ref [12]
		70, 1.5	WHSV = 0.2 h^{-1}	100	71	2	27	41		
	Pd/SBA-15	70, 1.5	WHSV = 0.5 h^{-1}	75.7	58.6	38.2	3.2	78		
	Pd/pyridyl	100, 3.6	n.a.	100	26	37 (DBI)	32	n.a.	Solvent-free	Ref [13]
	Pd/C	100, 3.5	6.3	100	19	49	30	170	H_2O was added; solvent-free	Ref [14]
	Pd/Al$_2$O$_3$	50, 2	4	13.4	95.9	0.9	3.2	34	H_2O/supercritical CO_2 biphasic solvent	Ref [15]
	Pd/Al$_2$O$_3$	80, 1	0.71	50	94	0.8	5.2	370 (*2800)	TOL was the major by-product	Ref [16]
	Pd/C	80, 1	n.a.	50	81	0	19	n.a.		
	Pd/C	60, 4	0.33	50	31	n,a,	26	218		Ref [17]
		60, 4	1.25	100	0	0	100	218		

	Pd/η-Al_2O_3	90, 1.5	4	100	91	6	3	41		Ref [18]
		90, 1.5	18	100	85	3.5	11.5	41		
	Pd/C	60, 0.6	2.3	100	80	0	20	180	$NaH_2PO_4 \cdot H_2O$ was added; H_2O/di-chloromethane biphasic solvent	Ref [19]
		30, 0.6	7.5	100	94	0	6	55		
	Pd/TiO_2	30, 0.6	12.5	75	94	0	6	13		
	Pd/γ-Al_2O_3	30, 0.6	14	50	90	0	10	7		
	Pd/C	r.t., 0.34	2	100	59	41	No	50		Ref [20]
	Pd/C	50, 2	4	100	0	100	No	250 (*2980)	H_2O/supercritical CO_2 biphasic solvent	Ref [21]
	Pd/Al_2O_3	50, 2	4	46	50	50	No	24		
	Pd/MCM-41	50, 2	4	90.2	90.9	9.1	No	230 (*4150)		
Pt-based	Pt/C	105, 5.5	8.6	94	5	81	3	212	Solvent-free	Ref [14]
		105, 5.5	2.5	100	0	97	3	780	H_2O was added; Solvent-free	
	Pt/C	r.t., 0.34	4	100	0	93	7	50		Ref [20]
	Pt/Al_2O_3	70, 1.5	WHSV = 0.5 h^{-1}	94.6	2.5	75.5	8.3	194	Trickle-bed reactor; WHSV = 0.5 g g_{cat} h^{-1}	Ref [12]
	Pt-Sn/SiO_2	60, 0.4	4	100	11	75	14	70		Ref [22]
	Pt-Sn/nylon	60, 0.1	2	50	15	80	2	58		Ref [23]
	Pt/nylon	60, 0.1	n.a.	100	10	83	4	n.a.		
	Pt NWs	80, 0.1	24	100	n.a.	95.4	n.a.	8.3		Ref [24]
	Pt/Al_2O_3	100, 1.5	24	100	12	68 (DBI)	n.a.	20		Ref [25]
	Pt/Ni-MOF	100, 0.8	10	92	1	99 (DBI)	No	18		Ref [26]
	Pt/C	50, 2	4	8.5	0	100	No	8		Ref [21]

	Pt/MCM-41	50, 2	4	20.6	19.8	80.2	No	97	H_2O/supercritical CO_2 biphasic solvent	
Rh-based	Rh/C	110, 8.3	5.3	100	22	42	1	380	H_2O was added; Solvent-free	Ref [14]
	Rh/Al_2O_3	70, 1.5	WHSV = 0.5 h^{-1}	99.8	16.6	77.1	1.5	102	Trickle-bed reactor; WHSV = 0.5 g g_{cat} h^{-1}	Ref [12]
	Rh/C	r.t., 0.34	8	100	0	100	No	13		Ref [20]
	Rh/polymer	r.t., 2	1	83	0	100 (DBI)	No	130	Rh complex stabilized on polymer	Ref [27]
	Rh/C	50, 2	4	28.8	25	75	No	14.4	H_2O/supercritical CO_2 biphasic solvent	Ref [21]
	Rh/Al_2O_3	50, 2	4	21	22.4	77.6	No	10		
Others	Ir/Al_2O_3	100, 2.1	n.a.	20	36	52	12	66		Ref [11]
	$Ru/K\text{-}Al_2O_3$	60, 0.1	82	100	93	n.a.	n.a.	1.2		Ref [28]
	Ru-NPs	290, 6.2	72	100	56.6	n.a.	n.a.	140		Ref [29]

n.a.: data not available; r.t.: room temperature; N.D. : non-detected.

† TOFs was calculated based on experimental results from the corresponding literature according to the following equation:

$$TOFs = \frac{moles\ of\ nitrile\ converted}{moles\ of\ total\ noble\ metal\ atoms\ \times reaction\ time}$$

***** Initial TOFs reported in literatures.

Supplementary Table 3 | Catalytic performance of the Pd/SiO_2 catalyst in BN hydrogenation with different reaction conditions.

Entry	Temperature (°C)	Reaction time (h)	Conversion (%)	Selectivity (%)			TOF (h^{-1})[d]
				TOL	BA	DBA	
1[a]	80	2	28.3	21.2	73.7	5.1	64
		9	95.6	25.2	66	8.7	
2[a]	70	2	13.0	15.5	76.7	7.8	31
		18	92.0	22.1	69.8	8.1	
3[b]	60	2	13.4	12.3	78.3	9.4	15.9
		20	93.0	13.2	75.6	11.2	
4[c]	80	2	23.0	20.2	75.2	4.6	54
		13	97.0	23.8	72.4	3.8	

[a] Reaction conditions: Solvent, 60 mL ethanol; BN, 0.5 g; catalyst, 30 mg; H_2 pressure, 0.6 MPa;
[b] Reaction conditions: Solvent, 60 mL ethanol; BN, 0.5 g; catalyst, 60 mg; H_2 pressure, 0.6 MPa;
[c] Reaction conditions: Solvent, 20 mL ethanol; BN, 0.5 g; catalyst, 30 mg; H_2 pressure, 0.6 MPa;
[d] TOFs were evaluated after proceeding the reaction for 2 h.

Supplementary Table 4 | Catalytic performance of the 5Pd-Ni/SiO_2 catalyst in BN hydrogenation with different reaction conditions

Entry	Temp. (°C)	H_2 pressure (MPa)	Reaction time (h)	Conversion (%)	Selectivity (%)			TOF (h^{-1})
					TOL	BA	DBA	
1	80	0.6	1	29.5	N.D.	2.8	97.2	515
			3	99.5	N.D.	3.5	96.5	
2	80	1.0	1	36.0	N.D.	5.3	94.7	630
			2.5	97.9	N.D.	7.9	92.1	
3	100	0.6	1	53.5	N.D.	11.8	88.1	935
			2	98.0	trace (<0.5)	13.5	86.0	
4	120	0.6	0.5	60.3	trace (<0.3)	15.9	83.8	2109
			1	99.3	trace (<0.7)	19.3	80.0	

Reaction conditions: Solvent, 60 mL ethanol; BN, 0.5 g; catalyst, 30 mg. TOFs were evaluated after proceeding the reaction for 1 h.
N.D. means “non-detected”.

Supplementary Table 5 | Catalytic performance of the 5Pd-Ni/SiO_2 catalyst in BN hydrogenation with different solvents.

Entry	Solvent	Reaction time (h)	Conversion (%)	Selectivity (%)			TOF (h^{-1})
				TOL	BA	DBA	
1	ethanol	1	29.5	N.D.	2.8	97.2	515
		3	99.5	N.D.	3.5	96.5	
2	methanol	1	28.0	N.D.	6.4	93.6	490
		3	95.2	N.D.	7.4	92.6	
3	isopropanol	1	15.3	trace (<0.6)	2.4	97.0	134
		9	97.1	trace (<0.7)	4.4	94.9	
4	n-hexane	1	6.9	N.D.	0.5	99.5	60
		12	67.8	N.D.	1.0	99.0	
5	dichloromethane	1	2.8	trace (<0.6)	0.6	98.8	24
		12	26.3	trace (<0.9)	1.2	97.9	

Reaction conditions: Solvent, 60 mL; BN, 0.5 g; catalyst, 30 mg; H_2 pressure, 0.6 MPa; temperature, 80 °C. TOFs were evaluated after proceeding the reaction for 1 h.
N.D. means “non-detected”.

Supplementary Table 6 | Catalytic performance of the 1Pt-Ni/SiO_2, 3Pt-Ni/SiO_2 and Pt/SiO_2 catalysts in BN hydrogenation.

Entry	Catalyst	Reaction time (h)	Conversion (%)	Selectivity (%)				TOF (h^{-1})
				TOL	BA	DBI	DBA	
1	1Pt-Ni/SiO_2	1	14.0	N.D.	7.8	89.7	2.5	1110
		6	95.2	N.D.	9.1	77.6	13.3	
2	3Pt-Ni/SiO_2	1	15.1	N.D.	0.4	94.5	5.1	517
		6	98.1	N.D.	15.5	70.3	14.2	
3	Pt/SiO_2	1	16.0	9.2	22.3	-	68.5	127
		9	99	11.1	16.1	-	72.8	

Reaction conditions: Solvent, 60 mL ethanol; BN, 0.5 g; catalyst, 30 mg, reaction temperature, 80 °C， H_2 pressure, 0.6 MPa. TOFs were evaluated after preceding the reaction for 1 h.
N.D. means “non-detected”.

Supplementary Table 7 | EXAFS data fitting results of *x*Pd-Ni/SiO_2 catalysts (*x* = 5, 10, 20) at Pd *K*-edge.

Sample	Path	N	R (Å)	σ^2 (10^{-3}Å^2)	ΔE_0 (eV)	*R*-factor
5Pd-Ni/SiO_2	Pd-Ni	5.5	2.54	7.6	3.3	0.004
	Pd-Pd	1.2	2.71	5.5	3.9	
10Pd-Ni/SiO_2	Pd-Ni	4.3	2.53	7.3	1.2	0.001
	Pd-Pd	3.6	2.72	7.0	3.4	
20Pd-Ni/SiO_2	Pd-Ni	2.7	2.53	7.6	1.0	0.001
	Pd-Pd	6.6	2.73	7.0	4.8	

Supplementary Notes

Supplementary Note 1

For Pd foil, two characteristic peaks ***A*** (24367 eV) and ***B*** (24391 eV) were clearly observed (Supplementary Fig. 10) On 5Pd-Ni/SiO_2 sample, these two peaks were much less pronounced and shifted to higher energies by about 1 ~ 2 eV, implying the high dispersion of Pd atoms and charge transfer between Pd and Ni, in well agreement with DRIFS CO chemisorption, and in situ XPS results (Fig. 3). As increasing the Pd coverage by varying the number of Pd ALD cycles from 5 to 20, the two characteristic peaks developed considerably and became similar to those of Pd foil on 20Pd-Ni/SiO_2 in shape, implying the aggregation of Pd from isolated atoms or tiny aggregates to continuous Pd islands or even Pd films. This result again agrees well with the DFIFTS CO chemisorption results (Fig. 3b).

Supplementary Note 2

Before introducing CO, the samples were in-situ reduced in 10% H_2/Ar for 1 h at 150 ºC for Pd/SiO_2 and 5Pd-Ni/SiO_2, and at 300 ºC for Ni/SiO_2, respectively. After exposing to 10% CO/Ar until saturation at room temperature, the samples were purged with high purity Ar at a flow rate of 20 mL/min for 30 min to remove the gas phase CO and an IR spectrum was then collected. After that the sample was further purged with 10% O_2 in Ar for another 30 min to remove the CO adsorbed on metallic Ni surface, and another IR spectrum was collected.

It is well-known that CO adsorbs on metallic Pd and Ni surface in very similar manner strongly at room temperature, which make the differentiation of the individual features of CO on Pd from Ni to be very challenging[30-32]. Here, after CO exposure, we employed additional O_2 purging to remove chemisorbed CO on Ni, so that the CO on Pd can be differentiated individually. This is because that the affinity of Ni surface with oxygen is greater than that with CO[33,34], so that gaseous O_2 would oxidize the Ni surface and readily displace the pre-adsorbed CO or remove the CO by forming CO_2[34,35]. In contrast, CO bonds to Pd more strongly than O_2, thus the pre-adsorbed CO on Pd keeps intact during O_2 purging[36].

As shown in Supplementary Fig. 13, on Pd/SiO_2, there were two main peaks centered at 2094 cm^{-1} and 1990 cm^{-1} after 30 min Ar purging, which were assigned to linear-bonded CO and bridge-bonded CO on Pd, respectively[37,38]. The spectrum remained intact after further purging the sample with 10% O_2/Ar for another 30 min,

indication of that the O_2 can't replace or oxidized the CO on Pd surface at room temperature, which was due to the hindrance of adsorption and dissociation of O_2 on Pd surface by strongly bonded CO[36]. On Ni/SiO_2, there were two main peaks centered at 2037 cm^{-1} and 1915 cm^{-1} after Ar purging, which were assigned to linear-bonded CO and bridge-bonded CO on Ni, respectively[39,40]. In contrast to CO adsorption on Pd, these peaks were almost vanished after O_2 purging, along with a new peak centered at 2342 cm^{-1} appeared, indication of the formation of gaseous CO_2. Clearly, the chemisorbed CO molecules on Ni were totally removed by O_2 by forming CO_2 at room temperature, in good agreement with previous literatures[34,35].

On 5Pd-Ni/SiO_2, after Ar purging, there were four peaks centered at 2092 cm^{-1}, 2046 cm^{-1}, 1975 cm^{-1} and 1916 cm^{-1}, overlapped with each other, which are assigned to linear-bonded CO on Pd, linear-bonded CO on Ni, bridge-bonded CO on Pd and bridge-bonded CO on Ni, respectively. After O_2 purging at room temperature, the two peaks at 2046 cm^{-1} and 1916 cm^{-1} totally disappeared, while only leaving the two peaks at 2092 cm^{-1} and 1975 cm^{-1} present. In addition, the new peak corresponded to CO_2 species at 2342 cm^{-1} also appeared, verifying the removal of CO chemisorbed on the Ni sites of PdNi bimetallic catalysts.

In brief, these results provide solid evidence that O_2 purging at room temperature can effectively remove the adsorbed CO molecule on Ni sites by passivating the metallic Ni surface with oxygen while bring no changes to the adsorbed CO molecule on Pd sites, thus making differentiation of CO on the Pd sites of PdNi bimetallic catalysts in a much clear manner.

Supplementary Note 3

In order to hydrogenate the C≡N triple bond in benzonitrile to form benzylamine (BA), two sequential hydrogenation steps are involved: First, BN hydrogenates with two H atoms to form the benzylideneimine (BI) intermediate; Second, the BI intermediate hydrogenates with another two H atoms to generate the target product BA. Various possible reaction paths were considered as shown in following equations:

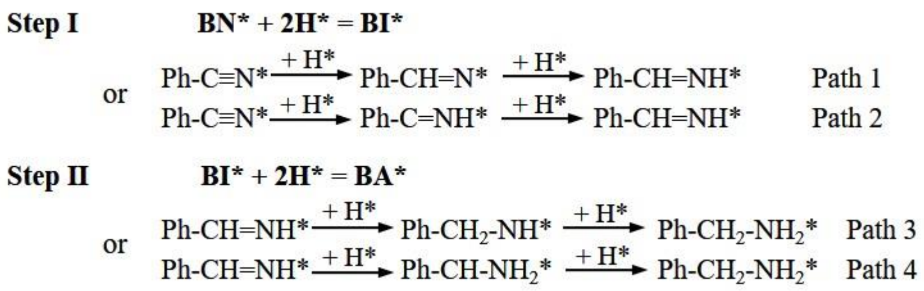

We noted that the first H atom attacking either C or N group during each hydrogenation step leads to different reaction energetics. In the hydrogenation step of BN to BI on Pd(111), the energy barriers for H atom attacking the C group first, then the N group are 1.21 and 1.17 eV, respectively (Path 1 in Supplementary Fig. 16a), which gives an effective barrier of 1.32 eV. On the other hand, the first H atom attacking the N group of BN followed by the second H attacking the C group to form BI (Path 2) has lower barriers of 0.84 and 0.98 eV (an effective barrier of 1.36 eV), respectively. While energetically, the first H atom attacking the C group in Path 1 is slightly endothermic by 0.15 eV, while the first H atom attacking N group in Path 2 is considerably endothermic by 0.38 eV. Therefore, H atom attacking the C group of BN first followed by N group to form BI (Path 2) is more favorable than the path 1. In the sequential hydrogenation step of BI to BA, the effective energy barrier of H atom first attacking the N group then the C group is 0.93 eV (Path 4), is also significantly lower than that of 1.43 eV for the path of H atom first attacking the C group then the N group (Path 3). These results suggest that subsequent hydrogenation of BI to BA prefers H atom attacking the N group first followed by attacking the C group.

The definition of effective energy barrier:

In this work, the effective energy barrier was used to evaluate the kinetics of adding one H_2 molecule in each individual hydrogenation step. It was defined as the energy threshold of conquering the two H atoms hydrogenation processes by taking both thermal dynamic and reaction kinetics (barrier) in individual steps account, as depicted in Supplementary Fig. 16b. In the process of hydrogenation of AB molecule into HABH, there are three possible situations, regarding the relative values of barriers in the two sequential hydrogenation steps and the relative energy levels of the intermediate state. In the case of the green profile, the effective energy barrier was defined as E_{eff} = E(TS2)-E(AB*+2H*); For the red pathway, the effective energy barrier

was defined as E_{eff} = E(TS1)-E(AB*+2H*); For the blue pathway, the effective energy barrier was defined as E_{eff} = E(TS2)-E(ABH*+H*+*).

Supplementary Note 4

In the two sequential hydrogenation steps of BN to BI then BA over Pt(111), the hydrogenation order of either the C group or the N group are also considered. In the first hydrogenation step of BN to BI, the energy barriers for H atom attacking the C group first, then the N group on Pt(111) are 1.05 and 0.92 eV, respectively (Path 1 in Supplementary Fig. 18), which gives an effective barrier of 1.05 eV. In sharp contrast, H atom attacking the N group first, then attacking the C group to form BI (Path 2) is more favorable with much lower barriers of 0.14 and 0.91 eV, respectively (an effective barrier of 0.91 eV). Similarly, in the sequential hydrogenation step of BI to BA, the effective energy barrier of H atom attacking the N group first then the C group is 1.20 eV (Path 4), is also lower than that of 1.27 eV for H atom attacking the C group first (Path 3). These results suggest that subsequent hydrogenation of BI to BA prefers H atom attacking the N group first followed by attacking C group in both steps.

Supplementary Note 5

In the two sequential hydrogenation steps of BN to BI then BA over Pd_1@Ni(111), the hydrogenation order of either the C group or the N group are also considered. In the first hydrogenation step of BN to BI, the energy barriers for H atom attacking the C group first, then the N group on Pd_1@Ni(111) are 1.18 and 1.56 eV, respectively (Path 1 in Supplementary Fig. 20), which gives an effective barrier of 1.56 eV. In sharp contrast, H atom attacking the N group first, then attacking the C group to form BI (Path 2) is more favorable with lower barriers of 1.06 and 1.06 eV (an effective barrier of 1.06 eV), respectively. Similarly, in the sequential hydrogenation step of BI to BA, the effective energy barrier of H atom attacking the N group first then the C group is 1.30 eV (Path 4), is also lower than that of 1.42 eV for H atom attacking the C group first (Path 3). These results suggest that subsequent hydrogenation of BI to BA prefers H atom attacking the N group first followed by attacking C group in both steps, very similar to the cases on Pd(111) and Pt(111) (Supplementary Figs. 16 and 18).

Supplementary Note 6

On Ni(111), we found that H atom first attacking the C group of BN followed by attacking the N group of the obtained intermediate to form BI is more favorable than the path with the opposite hydrogenation sequence (Path 1 in Supplementary Figure 23). The effective barrier is 1.22 eV. On the contrary, subsequent hydrogenation of BI to BA prefers H atom attacking the N group first followed by attacking the C group, and the effective barrier is 1.21 eV.

According to the knowledge learned on Pd(111) and Pt(111) (Fig. 4), the condensation reaction between BI and BA can be highly possible on Ni(111). In addition, we found that the BI intermediate adsorbs considerably stronger on Ni(111) (3.03 eV) than on Pd(111) (2.59 eV) and Pt(111) (2.08 eV) (Supplementary Figure 21). Such stronger adsorption would reduce the mobility of BI on Ni(111), thus further facilitating the condensation reaction.

Besides above, it is very interesting that hydrogenolysis of BA on Ni(111) has a much higher barrier of 1.71 eV, which would effectively inhibit the hydrogenolysis reaction on Ni(111), in agreement with the inhibition of toluene formation on the Pd_1Ni SASA catalyst.

Supplementary References

1 Bourgeat-Lami, E. & Lang, J. Encapsulation of inorganic particles by dispersion polymerization in polar media: 2. effect of silica size and concentration on the morphology of silica–polystyrene composite particles. *J. Colloid Interface Sci.* **210**, 281-289 (1999).

2 Wang, H. W., Wang, C. L., Yan, H., Yi, H. & Lu, J. L. Precisely-controlled synthesis of Au@Pd core-shell bimetallic catalyst via atomic layer deposition for selective oxidation of benzyl alcohol. *J. Catal.* **324**, 59-68 (2015).

3 Perdew, J. P., Burke, K. & Ernzerhof, M. Generalized gradient approximation made simple. *Phys. Rev. Lett.* **77**, 3865-3868 (1996).

4 Kresse, G. & Furthmuller, J. Efficient iterative schemes for ab initio total-energy calculations using a plane-wave basis set. *Phys. Rev. B* **54**, 11169-11186 (1996).

5 Kresse, G. & Furthmuller, J. Efficiency of ab-initio total energy calculations for metals and semiconductors using a plane-wave basis set. *Comput. Mater. Sci.* **6**, 15-50 (1996).

6 Blochl, P. E. Projector augmented-wave method. *Phys. Rev. B* **50**, 17953-17979 (1994).

7 Kresse, G. & Joubert, D. From ultrasoft pseudopotentials to the projector augmented-wave method. *Phys. Rev. B* **59**, 1758-1775 (1999).

8 Grimme, S., Antony, J., Ehrlich, S. & Krieg, H. A consistent and accurate ab initio parametrization of density functional dispersion correction (DFT-D) for the 94 elements H-Pu. *J. Chem. Phys.* **132**, 154104 (2010).

9 Monkhorst, H. J. & Pack, J. D. Special points for Brillouin-zone integrations. *Phys. Rev. B* **13**, 5188-5192 (1976).

10 Henkelman, G., Uberuaga, B. P. & Jonsson, H. A climbing image nudged elastic band method for finding saddle points and minimum energy paths. *J. Chem. Phys.* **113**, 9901-9904 (2000).

11 López-De Jesús, Y. M., Johnson, C. E., Monnier, J. R. & Williams, C. T. Selective hydrogenation of benzonitrile by alumina-supported Ir–Pd catalysts. *Top. Catal.* **53**, 1132-1137 (2010).

12 Dai, C. *et al.* Efficient and selective hydrogenation of benzonitrile to benzylamine: Improvement on catalytic performance and stability in a trickle-bed reactor. *New J. Chem.* **41**, 3758-3765 (2017).

13 Dines, M. B., DiGiacomo, P. M. & Callahan, K. P. Hydrogenation process utilizing novel catalyst. U. S. patent 4,384,981 (1983).

14 Greenfield, H. Hydrogenation of benzonitrile to dibenzylamine. *Ind. Eng. Chem. Prod. Res. Dev.* **15**, 156-158 (1976).

15 Yoshida, H. *et al.* A multiphase reaction medium including pressurized carbon dioxide and water for selective hydrogenation of benzonitrile with a Pd/Al_2O_3 catalyst. *Appl. Catal. A* **456**, 215-222 (2013).

16 Bakker, J. J. W., van der Neut, A. G., Kreutzer, M. T., Moulijn, J. A. & Kapteijn, F. Catalyst performance changes induced by palladium phase transformation in the hydrogenation of benzonitrile. *J. Catal.* **274**, 176-191 (2010).

17 McMillan, L. *et al.* The application of a supported palladium catalyst for the hydrogenation of aromatic nitriles. *J. Mol. Catal. A: Chem.* **411**, 239-246 (2016).

18 Dai, C. *et al.* The influence of alumina phases on the performance of Pd/Al_2O_3 catalyst in selective hydrogenation of benzonitrile to benzylamine. *Appl. Catal. A* **545**, 97-103 (2017).

19 Hegedűs, L. & Máthé, T. Selective heterogeneous catalytic hydrogenation of nitriles to primary amines in liquid phase: Part I. Hydrogenation of benzonitrile over palladium. *Appl. Catal. A* **296**, 209-215 (2005).

20 Rylander, P. N., Hasbrouck, L. & Karpenko, I. Coupling reactions of nitriles and anilines during hydrogenation. *Ann. N. Y. Acad. Sci.* **214**, 100-109 (1973).

21 Chatterjee, M. *et al.* Hydrogenation of nitrile in supercritical carbon dioxide: A tunable approach to amine selectivity. *Green Chem.* **12**, 87-93 (2010).

22 Gőbölös, S., Mahata, N., Borbáth, I., Hegedűs, M. & Margitfalvi, J. L. Hydrogenation of benzonitrile on Sn-Pt/SiO_2 catalysts prepared by introducing $SnEt_4$ to Pt/SiO_2: Role of tin. *React. Kinet. Catal. Lett.* **74**, 345-352 (2001).

23 Galvagno, S., Donato, A., Neri, G. & Pietropaolo, R. Liquid phase hydrogenation of benzonitrile over Pt and Pt-Sn catalysts. *J. Mol. Catal.* **58**, 215-225 (1990).

24 Lu, S. L., Wang, J. Q., Cao, X. Q., Li, X. M. & Gu, H. W. Selective synthesis of secondary

amines from nitriles using Pt nanowires as a catalyst. *Chem. Commun.* **50**, 3512-3515 (2014).
25 Paul, H., Basu, S., Bhaduri, S. & Lahiri, G. K. Platinum carbonyl derived catalysts on inorganic and organic supports: A comparative study. *J. Organomet. Chem.* **689**, 309-316 (2004).
26 Long, J., Yin, B., Li, Y. & Zhang, L. Selective hydrogenation of nitriles to imines over a multifunctional heterogeneous Pt catalyst. *AICHE J.* **60**, 3565-3576 (2014).
27 Mastrorilli, P., Rizzuti, A., Suranna, G. P. & Nobile, C. F. Supported rhodium catalysed hydrogenation reactions under mild conditions. *Inorg. Chim. Acta* **304**, 17-20 (2000).
28 Muratsugu, S. *et al.* Formation and nitrile hydrogenation performance of Ru nanoparticles on a K-doped Al_2O_3 surface. *Phys. Chem. Chem. Phys.* **17**, 24791-24802 (2015).
29 Ortiz-Cervantes, C., Iyañez, I. & García, J. J. Facile preparation of ruthenium nanoparticles with activity in hydrogenation of aliphatic and aromatic nitriles to amines. *J. Phys. Org. Chem.* **25**, 902-907 (2012).
30 Trenary, M., Uram, K. & Yates Jr, J. An infrared reflection-absorption study of CO chemisorbed on clean and sulfided Ni (111)—Evidence for local surface interactions. *Surf. Sci.* **157**, 512-538 (1985).
31 Dalmon, J.-A., Primet, M., Martin, G.-A. & Imelik, B. Magnetic and infrared study of CO chemisorption on silica supported nickel-copper alloys. *Surf. Sci.* **50**, 95-108 (1975).
32 Eischens, R., Francis, S. & Pliskin, W. The effect of surface coverage on the spectra of chemisorbed CO. *J. Phys. Chem.* **60**, 194-201 (1956).
33 Brennan, D., Hayward, D. & Trapnell, B. The calorimetric determination of the heats of adsorption of oxygen on evaporated metal films. *Proc. R. Soc. Lond. A.* **256**, 81-105 (1960).
34 Alexander, E. G. & Russell, W. W. Oxidation of carbon monoxide on thin films of nickel, palladium, and an alloy. *J. Phys. Chem.* **68**, 1614-1618 (1964).
35 Park, R. L. & Farnsworth, H. E. CO adsorption and interaction with oxygen on (110) nickel. *J. Chem. Phys.* **40**, 2354-2357 (1964).
36 Stephens, S. Surface reactions on evaporated pailadium films. *J. Phys. Chem.* **63**, 188-193 (1959).
37 Chen, Y. *et al.* Formation of monometallic Au and Pd and bimetallic Au–Pd nanoparticles confined in mesopores via Ar glow-discharge plasma reduction and their catalytic applications in aerobic oxidation of benzyl alcohol. *J. Catal.* **289**, 105-117 (2012).
38 Lear, T. *et al.* The application of infrared spectroscopy to probe the surface morphology of alumina-supported palladium catalysts. *J. Chem. Phys.* **123**, 174706 (2005).
39 Agnelli, M., Swaan, H. M., Marquez-Alvarez, C., Martin, G. A. & Mirodatos, C. CO hydrogenation on a nickel catalyst: II. A mechanistic study by transient kinetics and infrared spectroscopy. *J. Catal.* **175**, 117-128 (1998).
40 Layman, K. A. & Bussell, M. E. Infrared spectroscopic investigation of CO adsorption on silica-supported nickel phosphide catalysts. *J. Phys. Chem. B* **108**, 10930-10941 (2004).
